# Creating Designer Engineered Extracellular Vesicles for Diverse Ligand Display, Target Recognition, and Controlled Protein Loading and Delivery

**DOI:** 10.1002/advs.202304389

**Published:** 2023-10-22

**Authors:** Alena Ivanova, Lukas Badertscher, Gwen O'Driscoll, Joakim Bergman, Euan Gordon, Anders Gunnarsson, Camilla Johansson, Michael J. Munson, Cristiana Spinelli, Sara Torstensson, Liisa Vilén, Andrei Voirel, John Wiseman, Janusz Rak, Niek Dekker, Elisa Lázaro‐Ibáñez

**Affiliations:** ^1^ Discovery Biology Discovery Sciences BioPharmaceuticals R&D, AstraZeneca Pepparedsleden 1 Mölndal 431 50 Sweden; ^2^ Translational Genomics Discovery Sciences BioPharmaceuticals R&D, AstraZeneca Pepparedsleden 1 Mölndal 431 50 Sweden; ^3^ Advanced Drug Delivery Pharmaceutical Sciences BioPharmaceuticals R&D, AstraZeneca Pepparedsleden 1 Mölndal 431 50 Sweden; ^4^ Medicinal Chemistry Research and Early Development Cardiovascular, Renal and Metabolism BioPharmaceuticals R&D, AstraZeneca Pepparedsleden 1 Mölndal 431 50 Sweden; ^5^ Structure and Biophysics Discovery Sciences BioPharmaceuticals R&D, AstraZeneca Pepparedsleden 1 Mölndal 431 50 Sweden; ^6^ Clinical Pharmacology and Safety Sciences Sweden Imaging Hub BioPharmaceuticals R&D, AstraZeneca Pepparedsleden 1 Mölndal 431 50 Sweden; ^7^ Research Institute of the McGill University Health Centre Glen Site McGill University Montreal Quebec H4A 3J1 Canada; ^8^ DMPK Research and Early Development Cardiovascular, Renal and Metabolism BioPharmaceuticals R&D, AstraZeneca Pepparedsleden 1 Mölndal 431 50 Sweden; ^9^ Present address: Division of Radiotherapy and Imaging The Institute of Cancer Research London UK; ^10^ Present address: Myllia Biotechnology GmbH Am Kanal 27 Vienna 1110 Austria

**Keywords:** exosomes, extracellular vesicles, genetic engineering, protein loading, targeting

## Abstract

Efficient and targeted delivery of therapeutic agents remains a bottleneck in modern medicine. Here, biochemical engineering approaches to advance the repurposing of extracellular vesicles (EVs) as drug delivery vehicles are explored. Targeting ligands such as the sugar GalNAc are displayed on the surface of EVs using a HaloTag‐fused to a protein anchor that is enriched on engineered EVs. These EVs are successfully targeted to human primary hepatocytes. In addition, the authors are able to decorate EVs with an antibody that recognizes a GLP1 cell surface receptor by using an Fc and Fab region binding moiety fused to an anchor protein, and they show that this improves EV targeting to cells that overexpress the receptor. The authors also use two different protein‐engineering approaches to improve the loading of Cre recombinase into the EV lumen and demonstrate that functional Cre protein is delivered into cells in the presence of chloroquine, an endosomal escape enhancer. Lastly, engineered EVs are well tolerated upon intravenous injection into mice without detectable signs of liver toxicity. Collectively, the data show that EVs can be engineered to improve cargo loading and specific cell targeting, which will aid their transformation into tailored drug delivery vehicles.

## Graphical abstract

This study details a comprehensive cell engineering toolbox to genetically modify extracellular vesicles (EV) to enhance EV's ability to target cells of interest, load, and efficiently deliver protein cargo in recipient cells. The findings shed light on the potential of engineered EVs as advanced drug delivery vehicles for targeted and efficient protein delivery.

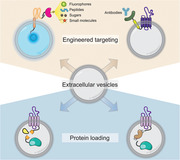

## 1 Introduction

Targeted delivery to desired sites of action is a major challenge for drug modalities such as enzymes, antibodies, peptides, and nucleic acids. The development of sophisticated drug delivery systems is therefore instrumental to improve the effectiveness of these biologicals. One potential solution is the use of extracellular vesicles (EVs), which can serve as a platform for drug encapsulation and have demonstrated efficacy in delivering therapeutics with proven clinical benefits.^[^ ^1^, ^2^ ^]^ These natural nano‐sized lipid‐bilayer particles transfer bioactive molecules that lead to functional responses and are involved in cell‐cell communication. They are released by almost all cell types and internalized by neighboring or distant recipient cells.^[^ ^3^ ^]^ Compared to synthetic nanoparticles, EVs offer unique advantages rendering them attractive alternative drug delivery systems. EVs have excellent biocompatibility, stability, and low immunogenicity.^[^ ^4^, ^5^ ^]^ They shield their cargo while in circulation^[^ ^6^ ^]^ and their surface provides naturally occurring sites for modifications that can contribute to their functionalization.^[^ ^7^ ^]^ These remarkable features are driving the advance of EV‐based therapies, but several challenges limit their therapeutic applications. These include rapid clearance from the circulation,^[^ ^8^ ^]^ inefficient intrinsic targeting that requires functionalization,^[^^9]^ and limited cargo loading capacity.^[^ ^10^ ^]^

Despite breakthroughs in EV engineering, delivery of EVs is often unspecific, and directing the cargo to specific cellular populations remains challenging.^[^ ^8^ ^]^ To address this problem, the EV surface has been extensively modified to display different types of targeting molecules that are recognized by specific cells.^[^ ^11^ ^]^ For example, antibodies or antibody fragments have been integrated into the surface of EVs.^[^ ^12^ ^]^ Since antibodies can be created against any chosen target, this approach to EV functionalization provides significant versatility. Nevertheless, the use of monoclonal antibodies as targeting moieties is currently limited due to their large size and complexity.^[^ ^13^ ^]^

Most cell engineering strategies for surface display focus on targeting peptides and proteins to EV protein sorting domains such as Lamp2b,^[^ ^14^ ^]^ tetraspanins,^[^ ^7^ ^]^ and PTGFRN,^[^ ^15^ ^]^ but the display of more complex molecules using this approach is challenging. An alternative strategy that has been understudied is EV surface modification using a cross‐linking reaction, known as azide‐alkyne cycloaddition or click chemistry to functionalize targeting moieties to the EV surface.^[^ ^16^ ^]^ This method is ideal for the introduction of macromolecules, small molecules, carbohydrates or polysaccharides to the surface of EVs via covalent bonds. It has previously been used to introduce targeting peptides such as an epidermal growth factor^[^ ^17^ ^]^ or peptides with high affinity to integrin αvβ3.^[^ ^18^ ^]^

Other important barriers prohibiting the full use of EVs as drug delivery vehicles are therapeutic cargo loading and cytoplasmic delivery within recipient cells. We and others have previously demonstrated that light‐ and small molecule‐inducible dimerization systems can be successfully used to increase the loading of EVs with cargo proteins such as SpCas9 and Cre recombinase.^[^ ^19^ ^]^ Although these technologies achieved delivery of functional proteins, the release of protein inside the EVs during the genetic engineering process is challenging and requires additional stimuli.

In this study, we have used multiple state‐of‐the‐art genetic engineering approaches to modify the composition of EVs in order to improve cell targeting and cargo loading. Specifically, we used these tools to decorate the surface of EVs with a vesicle anchor protein fused to a modified haloalkane dehalogenase protein tag (HaloTag). This tag is designed to covalently bind to synthetic ligands that harbor a chloroalkane linker^[^ ^20^ ^]^ and can therefore be used to introduce a variety of molecular effectors such as fluorophores, peptides, sugars, and small molecules on the surface of EVs. We used this system to decorate purified EVs with trivalent N‐acetylgalactosamine (GalNAc) and demonstrated that this results in preferential binding of the engineered EVs to primary human hepatocytes. We also developed a complementary system to display antibodies on the surface of EVs. Moreover, we utilized two different protein‐engineering approaches to improve the loading of Cre recombinase and protein release into the EV lumen during the genetic engineering process. These EVs deliver functional Cre into the cytoplasm of recipient cells upon the treatment with an endosomal escape enhancer. These modifications did not change the basic properties of EVs. The engineered EVs were well tolerated upon injection into mice and did not show detectable liver toxicity. Overall, our findings show that engineered EVs have considerable potential as unique targeted drug delivery systems for protein therapeutics.

## 2 Results

### 2.1 Generation and Characterization of EVs

**Figure 1:**
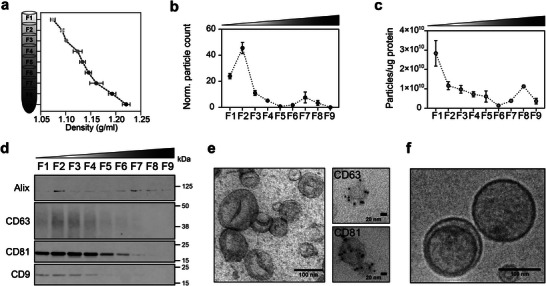
Generation and characterization of engineered EVs for cell targeting and protein loading. a) Schematic representation of Expi239F cell‐derived EV floatation on an iodixanol density gradient. Expi293F cell supernatant was collected and subjected to differential centrifugation. Small EVs (100k x *g* pellet) were subsequently bottom‐loaded in high‐resolution iodixanol density gradients (Optiprep) with decreasing densities (50%−10%, bottom to top). Nine fractions of 1 or 2 mL each were collected from top to bottom and their densities were analyzed by measuring the absorbance at 340 nm. b) Individual fractions (F1–F9) were analyzed using a nanoparticle tracking analyzer (NTA) to determine the particle number and c) the number of particles per µg of protein. Data are plotted as the mean of three independent experiments ± s.e.m. d) Representative western blot analysis of F1−F9 density fractions (12 µL/each lane). Membranes were blotted with the following antibodies: Alix, CD63, CD81, and CD9. Protein markers commonly associated with small EVs were enriched in the low‐density fractions. Based on the particle count, particle purity, and protein markers, fractions F1–F3 were pooled and considered as small EVs for further analysis. e) Representative negative staining TEM image of pooled fractions F1–F3 showing clean preparations of highly pure vesicles with a cup‐shaped morphology characteristic of the technique. Scale bar = 100 nm. On the right side, representative zoomed‐in immuno‐gold EM images of vesicles from low‐density fractions positive for CD63, and CD81 protein markers. Scale bar = 20 nm. Five microliters were loaded onto the grids. f) Cryo‐EM images of pooled EV samples (F1–F3) depicting EV size and morphology. Scale bar = 100 nm.

We produced engineered EVs in transiently transfected Expi293F suspension cells using the purification process previously described.^[^ ^8^ ^]^ Briefly, cellular supernatant was collected, and a differential centrifugation approach coupled with high‐resolution density gradient fractionation was used to isolate small EVs with a density of ≈1.10 g mL^−1^ (thereinafter referred to as EVs) (**Figure** **1**a). The highest particle count was measured in fractions F1‐F2, while most of the non‐vesicular components such as constituents of the conditioned cell culture media were present in fraction F7 (Figure 1b). Fraction F1–F3 showed the highest purity of vesicle based on the ratio of vesicle counts to protein concentration (Figure 1c). The presence of the canonical EV markers Alix, CD63, CD81, and CD9 in the gradient fractions confirmed the separation of EVs from higher‐density materials and protein aggregates (Figure 1d). Negative staining Transmission Electron Microscopy (TEM) of EVs revealed highly pure vesicle preparations of small size (<100 nm) and a round cup‐shaped morphology, typically observed using this technique^[^ ^21^ ^]^ (Figure 1e; Figure S1a, Supporting Information). Immuno‐gold labeling confirmed the presence of CD63+ and CD81+ vesicles (Figure 1e; Figure S1b, Supporting Information), in line with the western blot results. Cryo‐TEM analysis revealed electron‐dense vesicles with lipid bilayers and multi‐structural vesicles, such as double vesicles of small but heterogeneous size (Figure 1f; Figure S1c, Supporting Information). Overall, we conclude that fractions F1–F3 show the highest abundance and purity of EVs and we used these pooled EV fractions in all subsequent experiments.

### 2.2 CLIC1 is an Efficient Anchor Protein for EV Ligand Display

To select an efficient anchor protein for the display of targeting molecules, we studied the Cluster of Differentiation 47 (CD47) and Chloride Intracellular Channel 1 (CLIC1) proteins. These two proteins were chosen based on their differential capacities to facilitate the incorporation of cargo proteins into EVs. The selection process involved a comprehensive screening of EV protein databases with a focus on identifying proteins smaller than 50 kDa that could tolerate fusions without negatively affecting EV formation.^[^ ^7^ ^]^ Additionally, proteins were selected based on their ability to display ligands in the correct membrane topology orientation. We have previously shown that the macrophage “do not eat me” signal protein CD47 can be employed as an anchor protein for the display of EGFP on the surface of EVs.^[^ ^7^ ^]^ Here, we compare CD47 as an EV sorting domain, to the novel EV sorting domain CLIC1, a vesicle‐enriched protein that regulates various cellular processes such as cell volume, pH, membrane potential, and transmembrane transport.^[^ ^22^ ^]^ First, we generated fusion constructs of CD47 or CLIC1 with the enhanced green fluorescence protein (EGFP) and transiently transfected Expi293F cells to generate engineered EVs (Figure S2a, Supporting Information). The CLIC1‐EGFP fusion showed much higher expression levels in cell lysates and on EVs compared to CD47‐EGFP (Figure S2b, Supporting Information). Overexpression of neither of the fusion proteins affected the size distribution of the secreted EVs (Figure S2c, Supporting Information). To investigate the presence of EGFP on the surface of EVs, we used Proteinase K (PK) to degrade all the surface‐exposed proteins. The decreased signal or absence of EGFP and the Myc polypeptide protein Tag (Myc Tag) signals in the treated samples confirmed that EGFP was displayed on the surface of the vesicles (Figure S2d, Supporting Information). The levels of other transmembrane and intracellular proteins, which are typically enriched in EVs, such as Alix, CD63, TSG101, and β‐actin, remained generally stable after protease K treatment. This suggests that most of the EV particles remained intact despite the degradation of surface proteins. Based on the higher protein expression level, we decided to use CLIC1 as the basis for further modifications.

### 2.3 Display of Targeting Ligands on the Surface of EVs by HaloTag‐Based Conjugation

**Figure 2:**
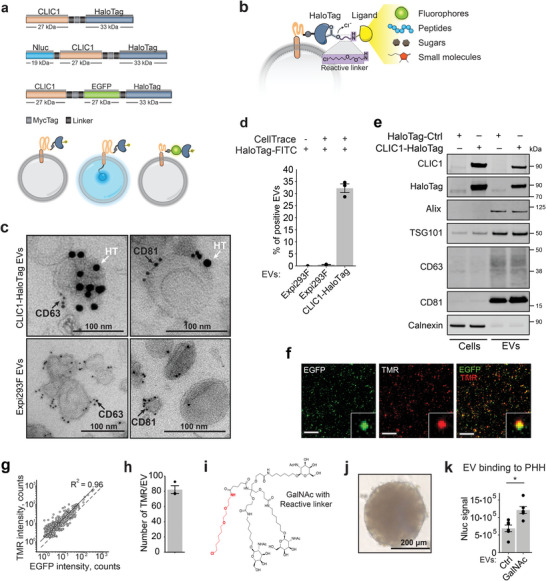
HaloTag enables a versatile and modular display of multiple targeting ligands on the surface of EVs. a) EV engineering construct design and the protein display topology within EVs. b) Schematic representation of the HaloTag designed to covalently bind various synthetic chloroalkane‐based targeting ligands. c) Immuno‐gold labeling of EVs collected from Expi293F cells transiently transfected with a plasmid coding for CLIC1‐HaloTag and naïve EVs as control. EVs were incubated with primary CD63 and CD81 antibodies followed by secondary antibodies conjugated with 6 nm gold particles (black arrow), and primary HaloTag antibody followed by a secondary antibody with 15 nm particles (white arrow). Scale bars = 100 nm. d) Nano‐flow cytometry analysis using the CytoFLEX system of EVs isolated from naïve Expi293F or HaloTag‐Myc‐CLIC1 transfected cells. HaloTag Ligand conjugated with Oregon green dye was used for HaloTag visualization. CellTrace Far Red Cell dye was used to label the whole EV fraction. Shown is an average of 3 independent experiments ± s.e.m. e) Representative western blot analysis of EVs from CLIC1‐HaloTag or mock‐transfected cells and corresponding cell lysates. f) EVs carrying EGFP and HaloTag were incubated with the HaloTag ligand – TMR and imaged on the surface of glass bottom plates. Shown are representative co‐localization images of EGFP and TMR fluorescent signals. Scale bar = 15 µm. g) Correlation of EGFP and TMR peak intensities counts for individual EVs from (f). The dotted line illustrates the 1:1 intensity ratio. Both EFGP and TMR co‐localize in the vague of observed particles. Over 850 EVs were analyzed in each of the 3 independent experiments. h) Quantification of the number of TMR molecules per EV. The peak intensity of the point‐spread function of each detected EV was extracted and divided by the single‐molecule signal to quantify the copy number of functional HaloTag molecules in each EV. Shown is an average of 3 independent experiments ± s.e.m. i) Chemical structure of the synthetic GalNAc derivate synthesized in‐house containing a HaloTag reactive chloroalkane linker (shown in red) for HaloTag biding and display on the EV surface. j) Representative image of the primary human hepatocyte (PHH) spheroids used as a human 3D liver model for the uptake experiments. Scale bar = 200 µm. k) Evaluation of the binding efficiency of engineered EVs to PHH. EVs display GalNAc at the surface and carry NanoLuc (Nluc) protein in the lumen for luminescent tracking. Shown is the average of luciferase signals from each spheroid (n = 6) in relative luminescence units ± s.e.m. The P‐value was calculated using a two‐sided Student's T‐test. p<0.05.

EVs were isolated from Expi293F cells transiently transfected with CLIC1‐HaloTag fusion constructs (**Figure** **2**a). Fluorescent EGFP and luminescent NanoLuc (Nluc) luciferase proteins and a Myc Tag were used in the construct design to characterize the EV engineering process and to track vesicles upon their addition to cells. In order to display interchangeable ligands on the EV surface, we fused CLIC1 to a HaloTag,^[^ ^20^ ^]^ which covalently binds to ligands that harbor a chloroalkane linker (Figure 2b). Negative staining TEM coupled with double immuno‐gold labeling using antibodies against EV protein markers conjugated with gold particles of different sizes demonstrated that both the CLIC1‐HaloTag fusion and the canonical vesicle‐associated markers CD63 or CD81 were present on the same vesicles (Figure 2c; Figure S2e, Supporting Information). These results were further supported by nano‐flow cytometry (Figure 2d; Figure S2f,g, Supporting Information). First, a CellTrace dye was used to label the whole EV fraction, as it binds to lysine residues and other amine sources, and next, EVs were stained with labeled antibodies against CD63, Myc Tag or an Oregon green Halo ligand for the HaloTag (Figure 2d; Figure S2f,g, Supporting Information). On average, 32% of the engineered EVs showed positive staining for the HaloTag (Figure 2d), and ≈17% of CD63 EVs were HaloTag^+^ (Figure S2g, Supporting Information). A marginally smaller proportion of EVs (≈25% and 12% for labeling with CFSE and CD63, respectively) were Myc Tag^+^, indicating a possibly lower labeling efficiency with the anti‐Myc tag antibody (Figure S2f,g, Supporting Information). Moreover, we confirmed the accurate surface positioning of the resulting HaloTag fusion protein through immuno‐gold EM labeling and flow cytometry analyses (Figure 2c,d and Figure S2e–g, Supporting Information). When these techniques were applied to engineered EV samples, only surface‐exposed proteins became available for antibody detection. Given that the EV integrity is maintained, and samples refrained from permeabilization, solely the HaloTag protein displayed on the EV surface was accessible for antibody detection. This approach solidified our validation of HaloTag's presence on the EV surface. EVs from CLIC1‐HaloTag or mock‐transfected cells and the corresponding cell lysates were also analyzed by western blotting for the presence of markers of endosomal origin that are expected to be enriched in EVs (Figure 2e). Compared to parental cell lysates, the CLIC1‐HaloTag fusion protein was highly enriched on the engineered EVs together with Alix, TSG101, CD63, and CD81 proteins, normally enriched on EVs.^[^ ^23^ ^]^ In contrast, we did not detect the endoplasmic reticulum (ER) protein marker calnexin, demonstrating the absence of ER‐associated intracellular vesicles in the preparations.

To determine whether the HaloTag protein can be used for the display of our targeting ligands, we incubated the recombinant HaloTag protein with a set of different chloroalkane reactive ligands. These included fluorophores (TMR, Oregon Green, Alexa Fluor 488), a peptide (GE11), an in‐house generated sugar (triantennary GalNAc), and the small molecule folate, all synthesized with reactive linkers. The protein‐ligand interactions were studied using mass spectrometry, which indicated that the process was exceedingly effective, with all ligands forming covalent connections with the HaloTag protein after a few hours of incubation (Table S1, Supporting Information).

Subsequently, to test the ability of the HaloTag to display ligands on the EV surface, we focused on the characterization of CLIC1‐EGFP‐HaloTag‐expressing EVs covalently conjugated with the non‐permeable fluorescent ligand TMR. The co‐localization of EGFP and TMR signals in EVs was evaluated by single‐molecule localization microscopy (SMLM). Most of the EGFP‐positive EVs carried the TMR ligand (Figure 2f,g) as demonstrated by the strong correlation between EGFP and TMR signal intensities (R^2^ = 0.96). SMLM enabled the quantification of the number and distribution of TMR and EGFP molecules per vesicle using reference standards (Figure S2h, Supporting Information) and showed that on average each EV carried ≈80 TMR molecules and a similar number of accessible HaloTag fusion proteins (Figure 2h). These results demonstrate that EVs are able to incorporate a CLIC1‐EGFP‐HaloTag fusion and that the HaloTag is functional and able to react with the fluorescent TMR ligand.

### 2.4 Surface Display of GalNAc on EVs Results in Improved Targeting to Primary Human Hepatocytes (PHH)

To explore the ability of HaloTag‐conjugated ligands to drive EV interactions with specific target cells, we focused on targeting hepatocytes, which have high expression levels of asialoglycoprotein receptor 1 (ASGR1). ASGR1 interacts with GalNAc, resulting in rapid internalization of the receptor‐ligand complex.^[^ ^24^ ^]^ We first de novo synthesized triantennary GalNAc with a reactive linker for covalent binding to the HaloTag (Figure 2i) and tested the binding efficiency in a ligand competition assay (Figure S3a, Supporting Information). EVs were labeled with the synthetically modified GalNAc followed by the addition of the TMR fluorescent ligand to evaluate vacant sites on the EV surface. Excess fluorescent ligand was removed through a size‐exclusion chromatographic resin and EV samples were analyzed by SMLM (Figure S3b, Supporting Information). GalNAc labeling for 1 h resulted in 40% of all available Halo sites being occupied in the EV preparation. Labeling efficiency further increased to 60% when the incubation was extended to 24 h. This indicated that GalNAc can efficiently be displayed on EVs (Figure S3b, Supporting Information). Using a human 3D liver model based on primary cells from one specific hepatocyte donor (Figure 2j),^[^ ^25^ ^]^ we showed that GalNAc conjugated EVs had a 1.8‐fold greater binding rate to PHH than non‐conjugated EVs expressing Nluc‐CLIC1‐HaloTag fusion proteins (Figure 2k). These results were confirmed with another hepatocyte donor (Figure S3c, Supporting Information). Together, these results demonstrate that GalNAc can be displayed efficiently on the surface of EVs by fusing CLIC1 as an anchor protein to a HaloTag and that the modification improves EV binding to human liver cells.

### 2.5 Antibody Binding Proteins Enable the Display of Antibodies on EVs

**Figure 3:**
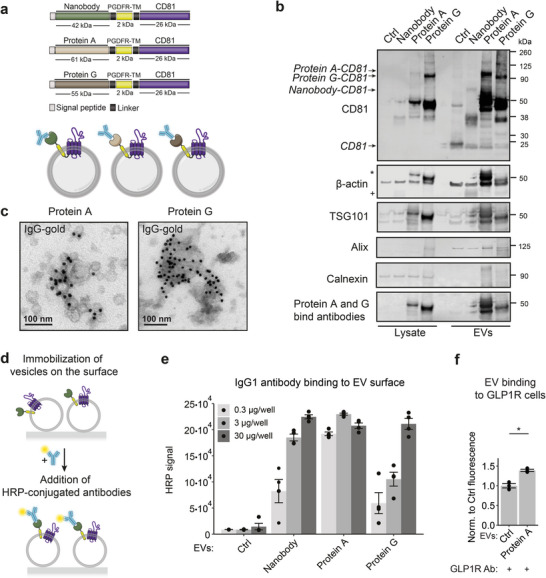
Display of antibody‐binding proteins on the surface of EVs. a) Schematic representation of the EV construct design and protein topology within EVs for the display of antibody‐binding proteins. b) Representative western blot analysis of EVs from cells transfected with Nanobody‐CD81, Protein A‐CD81, and Protein G‐CD81 or naïve cells and corresponding cell lysates. Asterisk indicates the binding of antibodies by proteins A and G. Cross marks bands from antibodies bound to Nanobody. c) Protein A‐CD81 and Protein G‐CD81 vesicles were incubated with IgG–gold antibodies and representative immuno‐gold EM images are shown. Both types of EVs have the binding capacity to IgG‐gold antibodies. Scale bar = 100 nm. d) Schematics representation of the EV immunoassay. EVs were first immobilized on the surface of Poly‐L‐Lysine coated plates, blocked to avoid unspecific binding of the antibodies to their surface, and incubated with HRP‐labeled antibodies for detection. The level of the HRP luminescent signal corresponds to the number of antibodies bound to the EV surface. e) EV surface binding of IgG1 antibody. EVs derived from cells transfected with Nanobody‐CD81, Protein A‐CD81, and Protein G‐CD81 or control (Ctrl) naïve cells (0.3, 3, or 30 µg of the protein) were immobilized followed by their incubation with IgG1‐HRP antibodies as described in (d). The average HRP signal in relative luminescence units ± s.e.m corresponding to the amount of bound IgG1 antibodies to the vesicles is shown (n = 4). No significant binding of IgG1 antibodies was shown in Ctrl vesicles. f) Evaluation of the binding efficiency of engineering EVs displaying anti‐GLP1 receptor (GLP1R) antibody to HEK293 cells overexpressing GLP1R. Protein A EVs or naïve EVs were first incubated with blocking solution followed by incubation with anti‐GLP1R antibodies, labeled with NHS ester Alexa Fluor 594 r dye. After washes, EVs were added to the cells for 1 h at 4 °C. Unbound EVs were washed out, and fluorescent signal was measured. Normalized to Ctrl average fluorescent signal from three biological replicates ± s.e.m. is shown. P‐value was calculated using a two‐sided Student's T‐test. Asterisks indicate that p<0.05.

Having established GalNAc‐dependent delivery of engineered EVs to hepatic cells, we next wanted to assess payload delivery to extrahepatic sites where retention of EVs is generally lower.^[^ ^8^ ^]^ Monoclonal antibodies can be effectively used to direct cargoes to cells of interest. Therefore, we developed strategies to decorate the surface of EVs with IgG class antibodies. Protein A, protein G and nanobodies all bind IgGs with high affinity and specificity.^[^ ^26^ ^]^ EVs were isolated from Expi293F cells transiently transfected with the three constructs with the right orientation for surface display, as schematically shown in **Figure** **3**a. We chose to display these proteins on the surface of EVs as a fusion with CD81, a marker protein enriched on EV surfaces.^[^ ^27^ ^]^ To achieve this, we introduced an additional transmembrane helix of the platelet‐derived growth factor receptor beta protein (PDGFRB)^[^ ^26^ ^]^ fused to the CD81 to allow the display of the fusion partner on the surface of the particle (Figure 3a). Western blot analysis revealed that protein A, protein G, as well as the nanobody fusions were abundant in the engineered EV fractions (Figure 3b). The antibody binding domains retained their IgG‐binding capacity during the western blot procedure reflecting high intrinsic stability against detergent denaturation. Next, we tested the presence of functional protein A, protein G, and nanobody molecules on the surface of EVs by incubating them with gold‐labeled IgGs, followed by TEM imaging (Figure 3c). EVs decorated with both protein A and protein G showed abundant binding of IgG‐gold‐labeled antibodies, suggesting that the vesicles were positive for the fusion proteins (Figure 3c; Figure S4a, Supporting Information). In contrast, we did not observe binding of IgG‐gold antibodies to nanobody‐carrying EVs, which could be a result of the TEM fixation procedure altering the function of the nanobody (Figure S4b, Supporting Information).

Then, we investigated if the engineered EVs maintain their antibody binding properties in the presence of a blocking solution that deposits a protein corona at the surface of the EVs, as previously observed for LNPs,^[^ ^28^ ^]^ and therefore potentially attenuate the antibody binding capacity. We used an enzyme‐linked immunosorbent assay (ELISA)‐based assay in which EVs were immobilized and incubated with a blocking solution to avoid unspecific binding of the antibodies (Figure 3d). Thereafter, horseradish peroxidase (HRP)‐coupled antibodies were added to estimate the antibody‐binding potential. Strikingly, all engineered EVs robustly bound HRP‐mAb conjugates. By titrating the amount of EVs from 0,3 to 30 µg per well, we demonstrated that protein A binds the IgG1 antibody isotype most effectively (Figure 3e). EVs without an antibody binding domain showed HRP levels comparable to the background signal, indicative of the absence of unspecific IgG binding. Thus, EVs displaying protein A on the surface were used for further validation of their targeting capacity.

Next, we investigated if these engineered EVs can direct vesicles to specific cell types through antibody‐mediated interactions. As a proof‐of‐concept, we chose targeted delivery to glucagon‐like peptide‐1 receptor (GLP1R)‐expressing cells. GLP1R is highly expressed in pancreatic beta‐cells^[^ ^29^ ^]^ but not in hepatic epithelial cells, and therefore this antigen might be used to study extrahepatic delivery of therapeutic EVs.^[^ ^30^ ^]^ Unmodified EVs or EVs containing the protein A‐CD81 fusion were labeled with the protein dye NHS ester Alexa Fluor 594 and incubated with both HEK293 cells that stably overexpressed GLP1R and wild‐type HEK293T cells. After 1 h at 4 °C, the EV‐associated fluorescence in recipient cells was 1.4‐fold higher for targeted EVs than for non‐targeted control EVs in HEK293 cells that stably overexpressed GLP1R, while no significant changes were observed in the HEK293T cells (Figure 3f; Figure S4c, Supporting Information). Together, these experiments demonstrated that specific antibodies can be displayed on the surface of EVs using anchor proteins such as protein A, protein G, and nanobodies. In the case of anti‐GLP1R antibody, the modification increased the binding of EVs to GLP1R overexpressing cells, and this interaction is driven by antibody‐receptor binding and not by the general stickiness of the modified EVs, highlighting the power of engineered EVs for targeting applications.

### 2.6 Development of Protein Engineering Systems for Efficient Cargo Loading into EVs

To improve protein loading and EV‐mediated cargo delivery to recipient cells, we developed two different protein loading systems. The first approach is based on a DnaB helicase (DnaBmini) Intein (Intervening Protein) derived from *Synechocystis sp*.,^[^ ^31^ ^]^ and the second uses a small molecule‐controllable TimeSTAMP tag (Time‐Specific Tagging for the Age Measurement of Proteins).^[^ ^32^ ^]^ These two protein engineering strategies were further developed to load Cre recombinase into the lumen of EVs by fusing it with the vesicle marker CD63.

The DnaB mini intein system combines a DNA helicase (DnaB) with an intein, which is a protein segment capable of self‐splicing. This reaction releases the intein from the precursor protein while ligating the flanking sequences, called exteins.^[^ ^33^ ^]^ External triggers such as changes in pH or temperature, lead to the spontaneous excision of the helicase domain and the subsequent release of the remaining protein fragment. However, many inteins display strong splicing in the absence of any cofactors. Because of these properties, intein technology is widely used as an important tool for protein purification, labeling, and engineering, enabling the study of protein structure‐function relationships and the purification of target proteins.^[^ ^31^ ^]^

**Figure 4:**
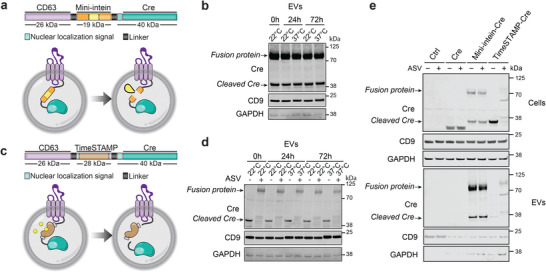
Protein loading of genetically engineered EVs. a) Schematic representation of the design of the DnaB mini‐intein fusion protein construct and the EV loading system. The tetraspanin CD63 (violet) was fused with a DnaB mini‐intein cassette consisting of a mini‐intein protein domain (yellow) and flanking extein (orange). The C‐term of the mini‐intein cassette was connected with Cre recombinase (green) with an N‐terminal nuclear localization sequence (light green) (NLS‐Cre). NLS‐Cre recombinase is then recruited in the EV lumen during biogenesis as a fusion with CD63. DnaB mini‐intein consists of the splicing domain and is modified to catalyze cleavage at its termini. Mini‐intein‐mediated cleavage reaction releases Cre recombinase from CD63‐Cre fusion protein. b) Representative western blot analyses of the Cre recombinase in EVs loaded with the DnaB mini‐intein system as a function of time and temperature. EVs isolated from Expi293F cells transfected with the mini‐intein loading system were incubated for 0, 24, or 72 h at 22 or 37 °C. GAPDH was used as a loading control and CD9 as an EV marker. c) Schematic representation of the design of a protein loading system with a TimeSTAMP (Time‐Specific Tagging for the Age Measurement of Proteins) drug‐controllable cargo release and loading in EVs. As above, between CD63 (violet) and NLS‐Cre recombinase (green) TimeSTAMP protein (brown) was inserted. The epitope tag (brown) is rapidly removed from the protein of interest by a sequence‐specific protease unless a protease inhibitor Asunaprevir (ASV) is present (shown in yellow). This approach allows the release of Cre recombinase from the fusion with CD63 protein in the EV lumen, once ASV is absent. d) Representative western blot analyses of the Cre recombinase EVs as a function of time and temperature. EVs were isolated from Expi293F cells transfected with the TimeSTAMP loading system. Cells were cultured with and without ASV (3 µm). EVs were incubated for 0, 24, or 72 h at 22 or 37 °C. GAPDH was used as a loading control and CD9 as an EV marker. e) Representative western blot analyses of cells and EVs secreted by Expi293F cells expressing the DnaB mini‐intein or TimeSTAMP loading systems. Cells were transfected with mini‐intein and TimeSTAMP constructs, plasmid overexpressing Cre recombinase or mock (Ctrl sample), and cultured with 3 M of ASV in indicated conditions. Cell supernatant was collected 48 h after transfection and subjected to differential centrifugation. Both loading approaches successfully brought cargo protein inside EVs. Levels of free Cre recombinase inside EVs can be estimated by the intensity of Cre protein bands ≈38 kDa. Bands ≈70 kDa correspond to CD63‐Cre fusion protein. GAPDH was used as a loading control and CD9 as an EV marker.

Here, we harnessed the DnaBmini intein split system to load protein cargo and facilitate its release within the lumen of EVs. For the first approach, we designed a fusion protein that consisted of CD63 vesicle sorting protein fused with the self‐cleaving SpDnaB mini intein [C1A], along with N‐extein and C‐extein protein splicing domains. Additionally, we incorporated Cre recombinase into the fusion protein, along with a nuclear localization signal. The fusion protein was designed to facilitate cleavage at the extein sites, resulting in the release of Cre recombinase from its fusion partner (**Figure** **4**a). By incorporating this fusion protein into the EVs as a fusion with CD63, we successfully loaded Cre recombinase into the vesicular lumen both as a fusion protein and as a cleaved protein, demonstrating efficient loading followed by the release of Cre. We tested several mutation variants of the mini‐intein system with Ser substitutions at position 155 (wt, S155D, S155V, and S155P) to modulate the C‐terminal cleavage activity.^[^ ^33^ ^]^ All mutants also contained the C1A substitution to block N‐terminal cleavage for the decrease in the full‐length fusion protein and the simultaneous increase of cleavage products. The presence of Asp at position 155 resulted in the best enrichment of free Cre in EVs based on the ratio of fusion protein versus cleaved Cre (Figure S5a,b, Supporting Information). Consequently, the mini‐intein variant S155D was used for the results shown in Figure 4. Interestingly, the Cre maturation experiment showed no further processing of the protein cargo post‐EV isolation over time or at different temperatures (Figure 4b).

In parallel, we explored the application of the TimeSTAMP tag as an alternative system for loading proteins and facilitating cargo release within EVs. The TimeSTAMP technique is based on a cell‐permeable drug‐controlled system designed for epitope tagging of newly synthesized proteins.^[^ ^32^ ^]^ In this method, a protein of interest is genetically fused with a cis‐acting protease and an epitope tag. Under normal conditions, the cis‐acting protease removes the epitope tag from the protein. However, if a specific protease inhibitor is present, the removal of the tag is prevented, and it remains attached to subsequently synthesized proteins. To inhibit cleavage and retain the epitope tag, we employed the protease inhibitor Asunaprevir (ASV).

By utilizing the TimeSTAMP tag system, we created a CD63‐TimeSTAMP‐Cre fusion protein, expressed it in Expi293F cells in the absence or presence of ASV, isolated the engineered EVs, and characterized the levels of Cre recombinase (Figure 4c). We successfully introduced the fusion protein consisting of the Cre recombinase, the cis‐acting protease, and the epitope tag into the EVs. Upon purification of EVs from the cells treated with ASV, we observe the whole fusion protein as a 100 kDa band. However, cells cultured without ASV secreted EVs where cleaved Cre protein was detected as a free protein in the lumen (Figure 4d). The cleavage kinetics of the protease appeared to be slow so that a moderate amount of the fusion protein persisted during EV biogenesis, followed by the release of Cre from the fusion partner. Interestingly, ASV not only blocked the protease activity in cells but also during the EV purification procedure as we barely detected cleaved Cre in this condition (Figure 4d). Overall, this approach allowed us to leverage the TimeSTAMP technique's ability to control epitope tagging and cargo release. Next, we tested if prolonged incubation of purified EVs can further increase the amount of cleaved Cre in EVs, but the levels remained stable over time independent of incubation temperature (Figure 4d).

Finally, we compared both systems by running side by side analysis (Figure 4e). A plasmid expressing Cre alone served as a control (Figure 4e, line 3, 4). In this condition, Cre was present in cell lysates and absent in EVs substantiating the need to use a system to actively escort Cre protein into EVs. The amount of fusion protein and cleaved Cre loaded into EVs by the mini‐intein system (Figure 4e, line 5, 6) was higher than that loaded by TimeSTAMP system (Figure 4e, line 7, 8). Overall, we conclude that both strategies work in principle, but that the SpDnaB mini intein system outperforms the TimeSTAMP approach based on the levels of cleaved Cre in EVs.

### 2.7 Cre Recombinase is Loaded into EVs as a Protein

To further assess whether delivery of Cre by engineered EVs is mediated through the transfer of proteins and not due to the plasmids used for transfections or mRNA being packaged into EVs, we generated a construct that included a drug‐controllable degradation tag on the CD63‐Mini‐intein‐Cre fusion protein (Figure S5d, Supporting Information). The Small Molecule‐Assisted Shutoff (SMASh) tag consists of a degron that removes itself in the absence of ASV.^[^ ^34^ ^]^ In the presence of ASV, the degron leads to the proteasomal degradation of the CD63‐Mini‐intein‐Cre fusion protein. In our study, cells transfected with the CD63‐Mini‐intein‐Cre‐SMASh construct and treated with ASV did not have detectable levels of Cre recombinase (Figure S5e, Supporting Information). No detectable levels of Cre recombinase we neither observed in EVs isolated from these engineered cells. These results confirm that the loading of the Cre recombinase in EVs is protein‐mediated in our experimental setup and is also in line with the observations that the expression of Cre from a plasmid is not sufficient for the detection of Cre protein in EVs (Figure 4e).

### 2.8 The Role of Endosomal Escape in the Delivery of Functional Cre Protein to Target Cells

**Figure 5:**
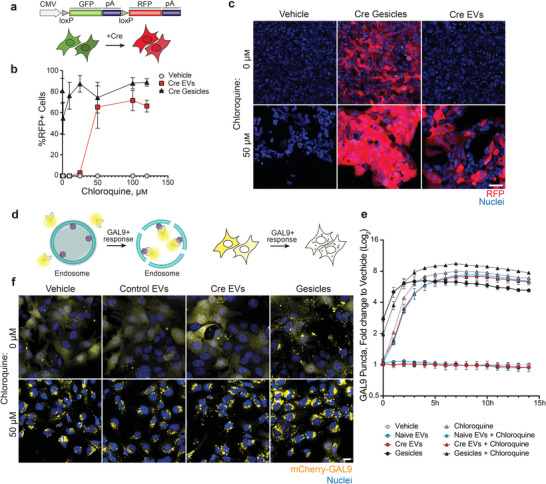
EV‐mediated functional delivery of Cre recombinase in reporter cells. a) HEK293 cell line expressing loxP‐GFP‐stop‐loxP‐RFP cassette. The CMV promoter initiates the expression of GFP, while the downstream RFP open reading frame (ORF) is inhibited by a stop codon located after the GFP ORF. Upon the presence of Cre protein in the nucleus, the DNA fragment between two LoxP sites is excised, and this enables the expression of the RFP ORF resulting in the fluorescent switch from GFP to the RFP in the cells. b) Image‐based quantification of the percentage of RFP positive cells following a 48 h co‐treatment with control EVs (without Cre recombinase), Cre recombinase loaded EVs via the DnaB mini‐intein system (Cre EVs), both at a concentration of 3.5 × 10^9^ EVs per well, and 0.5 µL commercial Cre recombinase Gesicles (Cre Gesicles, Takara) with indicated concentrations of chloroquine. Data is shown as an average of 3 independent experiments ± s.e.m. c) Representative image of the cells 48 h after the treatment described in (b) with 50 µM or 0 µM Chloroquine. Scale bar = 50 µm. d) Schematic overview of the Nanoprofiler Galectin‐9 (GAL9) reporter assay for endosomal escape evaluation. Cytosolic mCherry‐GAL9 recruits to the sites of endosomal damage via the binding of β‐galactoside sugar molecules. This results in the redistribution of the mCherry signal from the cytoplasm to endolysosome structures, giving the appearance of the mCherry puncta in the cells. e) Quantification of GAL9 puncta in HEK293‐GAL9 reporter cells treated with vehicle, naïve EVs, Cre EVs, Cre Gesicles, 50 µM of chloroquine, and a combination of naïve EVs, Cre EVs and Cre Gesicles with 50 µM of chloroquine. Cells were imaged at indicated times up to 14 h and GAL9 puncta were quantified. Data represents the average log2 fold change to the vehicle condition from three independent experiments ± s.e.m. f) Representative images of HEK293‐GAL9 cells 14 h after treatment described in (e). Scale bar = 50 µm.

To determine if EVs deliver functional Cre protein in vitro, we used a color switch HEK293 Cre reporter cell line.^[^ ^35^ ^]^ In these cells, Cre will trigger the excision of a loxP‐flanked GFP cassette and subsequent expression of RFP with a resulting color change from green to red (**Figure** **5**a). Reporter cells were treated with control EVs from untransfected Expi293F cells or Cre‐containing EVs derived from CD63‐mini intein‐Cre transfected cells. As a positive control, we used Gesicles (Takara Bio), commercial cell‐derived nanovesicles, which contain and deliver Cre recombinase protein and are decorated with the fusogenic vaccinia stomatitis virus G protein (VSVG) to aid fusion with the endosomal membrane and facilitate endosomal escape. Additionally, cells were treated with different concentrations of the endosomal escape enhancer chloroquine. Confocal imaging revealed that the addition of Cre Gesicles resulted in a robust and highly efficient delivery of Cre recombinase to target cells, with up to 90% recombination regardless of the presence of chloroquine (Figure 5b,c; Figure S6a,b, Supporting Information). In contrast, intein‐mediated Cre‐loaded EVs induced recombination in the reporter cells only in the presence of chloroquine, with an optimal efficacy and safety concentration of the drug at ≥ 50 µm (Figure 5b,c; Figure S6a,b, Supporting Information). These results suggest that pharmacological activation of endosomal escape in recipient cells is important for successful EV‐mediated transport and functional release of proteins.

To better understand the endosomal cargo release by engineered EVs, we employed our in‐house generated Nanoprofiler Galectin‐9 (GAL9) reporter assay designed to detect endosomal remodeling events.^[^ ^36^ ^]^ The GAL9 assay relies upon cytosolic mCherry‐GAL9 recruitment to the sites of endosomal remodeling through binding to exposed β‐galactoside moieties enriched in the endosomes. The resulting redistribution of the mCherry signal from the cytoplasm to endolysosome structures can be visualized as discrete mCherry puncta (Figure 5d). We treated mCherry‐GAL9 expressing reporter cells with chloroquine at concentrations ranging from 1 to 120 µM, and followed the formation of mCherry‐GAL9 puncta as a function of time up to 12 h (Figure S6c, Supporting Information). Maximal mCherry‐GAL9 responses were observed at chloroquine concentrations ≥ 50 µM 3 h post‐treatment. We quantified mCherry‐GAL9 puncta in HEK293‐GAL9 reporter cells that were treated with naïve EVs (EVs without Cre recombinase), Cre EVs (EVs carrying Cre recombinase protein loaded with the mini‐intein system), or a combination of naïve EVs or Cre EVs and 50 µM of chloroquine over 14 h (Figure 5e,f). We utilized Cre Gesicles and Cre Gesicles with 50 µM chloroquine as a positive control. Naïve EVs as well as Cre EVs did not induce the formation of mCherry puncta in the absence of chloroquine treatment (Figure 5e,f), but demonstrated similar kinetics and induction of mCherry‐GAL9 structures following chloroquine addition. In contrast, Cre Gesicles were capable of delivering Cre recombinase in the absence of chloroquine (Figure 5b,c). Interestingly, cells co‐incubated with Cre Gesicles and 50 µM chloroquine demonstrated an increased formation of mCherry‐GAL9 structures in comparison to the samples treated with Cre Gesicles alone, suggesting a synergistic or additive effect of the plasma fusion of the Gesicles and the action of chloroquine. Altogether, these results suggest that EVs are unable to elicit endosomal escape in the absence of pharmacological disruption of the endosome compartment regardless of carrying high levels of free protein cargo within their lumen and that endosomal remodeling, achieved via chloroquine co‐dosing, is necessary for efficient delivery of their cargo.

### 2.9 Engineered EVs Accumulate in Liver Macrophages without Observable In Vivo Liver Toxicity

**Figure 6:**
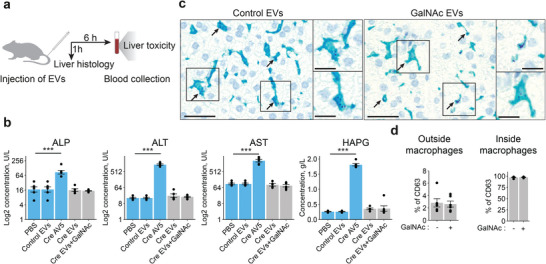
Engineered EVs have a safe liver toxicity profile in vivo. a) Schematics for the experimental design. EVs (1 × 10^11^ particles) or AV5 (1.4 × 10^9^ PFU) were injected into the tail vein of mice. Liver tissues were collected 1 h after injection for histology analysis. Blood from the saphenous vein was collected 6 h after particle tail vein injection for toxicity analyses. b) The levels of selected liver toxicity markers were compared among PBS control animals and control EVs (without Cre recombinase), adenovirus 5 with Cre recombinase protein (Cre AV5), EVs carrying Cre recombinase (Cre EVs), EVs carrying Cre recombinase and decorated with GalNAc molecules (Cre EVs+GalNAc) (*n* = 6). The average values indicated for each panel parameter ± s.e.m. are shown. The P‐value was calculated using a two‐sided Student's T‐test. Significant differences are indicated by asterisks (*** = *p*<0.001). c) Representative liver histology images of the animals treated with HaloTag engineered EVs with or without GalNAc. An anti‐human CD63 antibody assay was developed to detect injected particles in the tissue (purple dots) 1 h after the treatment. Kupffer cells were stained with F4/80 antibodies (bright blue). Arrows indicate the accumulation of CD63 signals inside cells. Scale bar = 50 µm in the wide‐field images and 25 µm in the magnifications. d) Quantification of the results described in (c). For both treatment conditions, most of the CD63‐positive cells were liver macrophages (Kupffer cells) (right panel). The average percentage ± s.e.m. of CD63‐positive cells of other types (CD63 signal is outside macrophages) is indicated on the left panel. Each data point represents different liver sections (*n* = 6).

In a previous study, we established that naïve EVs derived from Expi293F cells possess a favorable safety profile when administrated in vivo.^[^ ^5^ ^]^ In the current study, we sought to investigate the safety of specific modifications made to these engineered EVs. We intravenously (*i.v*.) injected HaloTag EVs with or without GalNAc targeting ligand into mice and evaluated liver toxicity by blood chemistry and histology analysis (**Figure** **6**a). Naïve EVs were used as negative control and adenovirus 5 (AV5) was used as a positive control.^[^ ^37^ ^]^ We collected blood 6 h after *i.v*. injection and evaluated liver toxicity by measuring blood levels of alkaline phosphatase (ALP), alanine aminotransferase (ALT), aspartate aminotransferase (AST), and haptoglobin (HAPG) as standard biomarkers for liver damage. While AV5 injection caused elevation of all measured markers, no substantial elevation was observed for any of the EV‐treated mice groups, which showed levels comparable to the PBS control (Figure 6b). These results demonstrate that HaloTag engineered EVs are not associated with any apparent liver toxicity. Immunostaining of collected liver tissues 1 h post‐EV administration using an anti‐human CD63 antibody to track EVs, showed that despite the introduction of GalNAc, over 97% of the injected EVs were phagocytosed by Kupffer cells, which are resident liver macrophages (Figure 6c,d). This is in line with previous reports that demonstrated active uptake of EVs by macrophages in the liver.^[^ ^38^ ^]^ In conclusion, while the overall safety profile of engineered EVs is encouraging, the entrapment of therapeutic EVs in Kupffer cells will need to be addressed to achieve specific cell targeting.

## 3 Discussion

One of the biggest challenges for the development of effective and safe therapies is targeted delivery. Side effects associated with immunogenicity, tissue barriers, off‐target delivery, endosomal escape, and rapid elimination, continue to impede the development of improved drug delivery systems.^[^ ^39^ ^]^ In this regard, EVs are promising alternatives that might help to overcome some of the difficulties.^[^ ^6^, ^7^, ^40^ ^]^ In this work, we have used state‐of‐the‐art EV engineering strategies to address two major bottlenecks in EV research: cell‐type specific targeting and efficient cargo loading. Surface display of distinct targeting ligands, in our case triantennary GalNAc molecule and anti‐GLP1R antibodies, was achieved through the expression of an EV sorting domain fused to either the HaloTag or to protein A. In addition, we developed new strategies to efficiently load protein cargo into EVs and to enrich the free form of this cargo inside the vesicles for EV‐mediated intracellular delivery.

Different ways to modify the EV surface composition by engineering anchor proteins have been explored with the aim of directing the vesicles to target cells.^[^ ^7^, ^14^, ^40^ ^]^ After screening a set of candidate proteins,^[^ ^7^ ^]^ we discovered that CLIC1 was the most promising candidate for surface target display. Our genetically modified versions of CLIC1 were abundant in EVs, making them an attractive and versatile candidate for the display of different molecules. While a wide range of peptides and proteins have been successfully displayed on the EV surface,^[^ ^1^ ^]^ the introduction of a polyvalent ligand that allows the plug‐and‐play display of multiple distinct types of molecules has not been explored. One of the major innovations of this study is the implementation of the HaloTag strategy as a universal tool for multi‐ligand EV surface decoration. The HaloTag enables the surface display of any type of “homing” molecule that carries a reactive linker for the conjugation to the tag.^[^ ^20^ ^]^ In this study, we used this system to display triantennary GalNAc on the surface of EVs, but other molecules, such as peptides and small molecules can be displayed as well. This exciting technology expands the possibilities for utilizing EVs as a versatile platform for targeted delivery using a wide range of established ligands.

We also engineered EVs to carry antibody‐binding domains for the decoration of EVs with any antibody. Prior research has explored other methods, for instance, the expression of genes encoding single‐chain variable fragments (svFv) of anti‐epidermal growth factor receptor (EGFR) and anti‐CD3 antibodies fused with the transmembrane domain of PDGFR^[^ ^41^ ^]^ or the use of lactadherin as an anchor to attach scFv fragments that have an affinity for human EGFR.^[^ ^42^ ^]^ However, incorporating entire antibodies into the EV surface using gene engineering approaches is challenging or even impossible due to the complex structure of antibodies.^[^ ^13^ ^]^ To overcome this limitation, alternative approaches were adopted, such as modifying EV surfaces with nanobodies. For example, Kooijman et al. mixed EVs with micelles containing polyethylene glycol and EGFR nanobodies for targeting EGFR overexpressing cancer cells.^[^ ^12^ ^]^ Another strategy involved clicking azide‐modified EVs with dibenzocyclooctyne‐derivatized SIRPα antibodies.^[^ ^43^ ^]^ Our approach did not involve fusing the antibody sequence directly to EV‐associated proteins or chemical modifications of antibodies, which could have been hampered by the size and complexity of the antibody structure. Instead, we facilitated the binding of full‐size GLP1R antibodies to the EV surface by incubating Protein A‐modified EVs with antibodies in solution. This method proved effective in enhancing the affinity of EVs for GLP1R‐expressing cells. This technique provides a powerful tool for screening a large number of EV‐antibody pairs for targeted delivery. Moreover, it enables quick functionalization of EV surfaces with unmodified antibodies, which expands the possibilities for utilizing EVs in targeted therapy applications.

In this study, we describe the use of different EV‐anchor proteins: CLIC1, CD81, and CD63 for luminal or surface display of protein cargo in EVs. By offering a diverse range of tagging options, we aimed to accommodate the display orientation, nature of the ligand, vesicle protein profile based on their cell source, and EV subpopulation preferences that inevitably arise in the field of EV‐mediated delivery. This strategic diversity ensures that our research provides a comprehensive toolkit that can be tailored to diverse research contexts and drug delivery applications.

Ensuring the specificity and selectivity of protein loading into EVs presents several challenges. EVs have a natural cargo sorting mechanism that favors certain types of proteins like tetraspanins.^[^ ^3^ ^]^ Overcoming this inherent bias and achieving selective loading of desired proteins is difficult, especially since the cargo loading process should not negatively impact the biogenesis, stability, or biological properties of the EVs. Addressing these challenges requires the development of innovative loading techniques. Here, we developed and compared two novel protein loading systems, the TimeSTAMP and DnaB mini‐intein. These methods are efficient at enriching protein cargo in EVs and enable the liberation of the cargo protein from the anchor protein inside the vesicles. Although both systems successfully recruited Cre recombinase, the DnaB mini‐intein‐based loading outperforms the TimeSTAMP system and achieves a higher amount of cleaved Cre in the EV lumen.

Regardless of efficient loading and release of protein within EVs, EV‐mediated delivery of protein cargo into cells is hindered by the low levels of endosomal escape. After internalization, the majority of EVs are trapped in endosomes and degraded or recycled in lysosomes, which complicates the delivery of an effector protein into the cytoplasm.^[^ ^44^ ^]^ We observed that a combination of targeting and treatment with an endosomal escape enhancer is required to achieve EV‐mediated functional delivery of protein cargo in recipient cells, reinforcing the idea that endosomal escape is a major bottleneck for the development of EV‐based protein delivery systems. To overcome the limited efficiency of EVs, many researchers are currently exploring the approach of overexpressing VSV‐G or other fusogenic viral‐like proteins on the surface of engineered EVs to facilitate in vivo performance and functional delivery of EV cargo.^[^ ^45^ ^]^ However, it is important to consider that VSV‐G and similar viral‐like proteins are highly immunogenic, which can restrict their therapeutic application, particularly for repeated in vivo dosing or chronic therapies.^[^ ^46^ ^]^

One of the concerns with engineered EVs is the possibility that the engineered tags or targeting ligands might cause immunogenicity in vivo. In this work, we show that, when injected intravenously in mice, engineered EVs have a good safety profile and do not induce detectable liver toxicity. However, they are prone to be digested by macrophages in the liver as previously reported,^[^ ^47^ ^]^ although a small percentage of the injected GalNAc‐decorated EVs reach liver hepatocytes. Separating GalNAc‐positive EVs from the mixture of EVs secreted by transfected cells through additional purification steps could potentially enhance the accumulation of these vesicles in hepatocytes. Even so, this tendency of EVs to accumulate in liver macrophages where they undergo lysosomal degradation ^[^ ^3^ ^]^ could potentially also be exploited therapeutically, for example for treating lysosomal storage disorders and modulating liver macrophages.^[^ ^48^ ^]^

Yet, to observe clinically relevant levels of cargo delivery, efforts beyond targeting modifications of EVs are needed. Avoiding the rapid elimination of nanoparticles from the bloodstream might help improve the delivery of the particles to target organs. It has been shown that the administration of compounds that deplete macrophages, such as clodronate liposomes^[^ ^49^, ^50^ ^]^ or gadolinium chloride,^[^ ^51^ ^]^ can substantially prolong nanoparticle circulation.^[^ ^49^, ^52^ ^]^ Additionally, similar effects on the circulation time of EVs were observed by blocking the function of mononuclear phagocytes by injecting large doses of organic and inorganic materials such as colloidal carbon,^[^ ^53^ ^]^ latex beads,^[^ ^54^ ^]^ dextran sulfate,^[^ ^55^, ^56^ ^]^ methyl palmitate^[^ ^57^ ^]^ or liposomes.^[^ ^54^, ^56^, ^58^ ^]^ Recently, progress has also been made in prolonging the half‐life of nanoparticles in the circulation by partial transient depletion of erythrocytes.^[^ ^59^ ^]^ While still in the experimental stage, these strategies hold promise for improving the pharmacokinetics and therapeutic efficacy of various nanoparticle‐based therapies. However, the achievement of low levels of associated toxicity is crucial for their successful implementation. Thus, the development of these different treatments for clinical applications could pave the way for better functional delivery of cargo using EVs.

## 4 Conclusion

In summary, we successfully engineered EVs to acquire enhanced specific targeting capabilities and to improve the loading of protein cargo. These advances make EVs a versatile and adaptable delivery platform with the potential for a wide range of applications.

## 5 Experimental Section

### Mammalian Cell Culture

Expi293F cells were cultured in a chemically defined Expi293 expression media (Thermo Fisher, A1435101) according to manufacturer recommendations. This media is protein‐free and serum‐free, and it has been developed to significantly reduce the risk of proteins co‐precipitating and interfering with protein expression experiments, which is particularly suited to limit the recovery of non‐EV material. Expi293F cells were transiently transfected at a 3.8‐4.2 × 10^6^ cells mL^−1^ confluency using PEI MAX 40 K (Polysciences Inc) as previously described.^[^ ^8^, ^60^ ^]^ After 24 h, cell viability was measured, and new Expi293 expression media was added to the cultures. 48 h post‐transfection, cells were collected, viability was measured, and if the cell viability exceeded 80%, cell supernatant was harvested for EV isolation. Expi293F cells were transiently transfected with different expression plasmids to facilitate a rapid and efficient screening of the constructs, to enable swift identification of optimal EV designs. A complete list of expression plasmids is available in Table S2 (Supporting Information).

In‐house generated HEK293 GLP1R overexpressing cells and unmodified HEK293T cells were cultured in high glucose DMEM, 2 mm glutamine, 10% fetal bovine serum (FBS), 0.1 mm MEM Non‐Essential Amino Acids at 37 °C in 5% CO_2_. HEK293‐loxP‐GFP‐RFP Cre recombinase stable cell line with a Puromycin selection marker (SC018‐Puro, Amsbio) and HEK293‐GAL9 reporter cells were cultured in high glucose DMEM, 2 mm glutamine, 10% FBS, 0.1 mm MEM Non‐Essential Amino Acids, and 5 µg mL^−1^ Puromycin (all from Gibco, Thermo Fisher Scientific) at 37 °C in 5% CO_2_. Before EV treatment, the Cre reporter cells were supplemented with FBS that was EV‐depleted by ultracentrifugation at 100.000 x *g* (Type 45 Ti, Beckman Coulter, Brea, CA) for 16 h at 4 °C and passed through a 0.22 µm filter (Merck Millipore). All cells were mycoplasma negative and authenticated by short tandem repeat DNA profiling analysis.

### Primary Human Hepatocyte (PHH) 3D Spheroid Culture

Cryopreserved PHH were purchased from BioIVT. Cells from two donors (SMC; female hepatocytes and OFA; male hepatocytes) were thawed according to the supplier's instructions and spheroids were formed as earlier described.^[^ ^25^ ^]^ Briefly, 2000 viable cells/well were seeded in ultra‐low attachment U‐bottom plates (Corning) in Williams E medium (PAN‐Biotech) supplemented with 5.5 mm glucose, 2 mm L‐glutamine (GlutaMAX), 100 U mL^−1^ penicillin, 100 µg mL^−1^ streptomycin, 0.1 nm insulin, 5.5 µg mL^−1^ transferrin, 6.7 ng mL^−1^ sodium selenite, 100 nm dexamethasone, and 10% FBS, and plates were centrifuged for 2 min at 100 x *g*. Between days 4–7, 50% of the medium was changed daily with FBS‐free medium, and spheroids were used for cell binding assays 8–25 days after seeding.

### Quantification of Cell Viability

Cell viability was measured using an automated cell counter system CEDEX (Innovatis GmbH) based on Trypan Blue exclusion method for determining cell viability. Cell viability was checked on the day of EV isolation and upon treatment of cells with ASV. For transiently transfected Expi293F cells, only cell‐conditioned media of cells with over 90% viability at 48 h were used for EV isolation experiments.

### Plasmid Design

Protein sequences of designed fusion proteins were reverse‐translated into a DNA sequence using codon optimization for protein expression in *Homo sapiens*. DNA fragments were synthesized at GenScript and cloned into the pEBNAZ expression vector using SacII and NotI restriction sites, under the control of the CMV promoter. Constructs Nanobody‐CD81‐EGFP, Protein A‐CD81‐EGFP, and Protein G‐CD81‐EGFP were assembled in‐house using Golden gate assembly in pcDNA3.1 vector.

### EV Isolation and High‐Resolution Iodixanol Density Gradient Fractionation

EV isolation and characterization were carried out according to the MISEV guidelines.^[^ ^61^ ^]^ Cells were first pelleted by centrifugation at 300 x *g* for 10 min, and cell debris and larger EVs were removed by centrifugation at 2500 x *g* for 30 min. The cell supernatant was transferred to 94 mL quick‐seal polyallomer tubes (Beckman Coulter) and centrifuged at 20000 x *g* for 25 min at 4 °C to pellet larger vesicles followed by transfer of the supernatant to new tubes and second centrifugation at 100 000 x *g* for 2 h at 4 °C (Type 45 Ti, k‐factor 210.4, Beckman Coulter) to pellet EVs. The EV‐enriched pellet was resuspended in 1 mL PBS further fractionated by flotation on iodixanol density gradients adapting the previously described protocol.^[^ ^8^, ^62^ ^]^ Briefly, A total of 1 mL of EV sample was mixed with 5 mL of iodixanol stock solution (Sigma–Aldrich) and laid at the bottom of the tube. Then 1 mL layers with increasing iodixanol density (35%−24%) were subsequently overlaid, forming a discontinuous gradient. For a higher resolution in the EV‐floating fractions, 2 mL layers of 22%, 20%, and 10% iodixanol were further overlaid. The gradient was centrifuged for 16 h at 120000 x *g* (SW 32.1 Ti, k‐factor 249.1, Beckman Coulter) at 4 °C and nine fractions were collected from the top to bottom. The density of each fraction was measured by absorbance at 340 nm using a PHERAstar FSX microplate reader (BMG Labtech). F1−F3 were pooled, transferred to new 94 mL PBS tubes, and ultracentrifuged at 120000 x *g* for 2.5 h (Type 45 Ti, k‐factor 175.3, Beckman Coulter). The EV pellets were resuspended in PBS and were freshly processed or stored at −80 °C.

### Protein Extraction and Immunoblot Analysis

Protein extraction and Western blotting from cell lysates and EV samples were performed as previously described.^[^ ^62^ ^]^ For PK treatment, EVs isolated from cells transfected with CLIC1‐EGFP were incubated with 2, 5, 10 or 20 µg mL^−1^ of Proteinase K for 30 min at 37 °C. After, the proteinase K was inactivated with 5 mM PMSF serine protease inhibitor for 10 min at room temperature (RT) followed by the lysis of EVs as described before.^[^ ^61^ ^]^ An equal amount of proteins or particles of each sample were mixed with sample buffer NuPAGE (Invitrogen) and heated at 70 °C for 5 min. Proteins were separated on 4%–12% SDS‐PAGE Bis‐Tris gels (Life Technologies) at 180 V in MES SDS running buffer and transferred using Trans‐Blot Turbo Mini or Midi polyvinylidene fluoride transfer packs (Bio‐Rad Laboratories, Hercules, CA, USA). The membranes were blocked with Odyssey TBS Blocking Buffer (LI‐COR Biosciences Inc, Lincoln, NE) for 1 h at RT with gentle shaking. After blocking, the membranes were incubated overnight at 4 °C with primary antibodies diluted in TBS Odyssey blocking buffer (Table S3, Supporting Information). The membranes were washed with 0.1% TBS‐Tween for 5 min, three times and incubated with the corresponding secondary antibodies for 1 h at RT, and then incubated for 1 h at RT with the following secondary antibodies diluted 1:20000 in 0.1% TBS‐Tween (Table S3, Supporting Information). Membranes were then washed three times for 5 min with TBS‐Tween, one time with PBS, visualized on the Odyssey CLx imaging system (LI‐COR), and analyzed with the Image Studio v.4.0.

### Particle Size and Concentration Measurements by Nanoparticle Tracking Analyzer (NTA)

Particle size and concentration of the EV samples were determined with NTA using a NanoSight instrument Malvern Instruments Ltd, Malvern, UK) equipped with an LM10 view unit and blue laser (405 nm, 70 mW) and NTA 2.3 analytical software. Samples were diluted in 0.22 µm filtered PBS and analyzed as follows: camera level 16, with 40–100 particles per frame, and acquisition time of 90 sec for three videos. Software settings were kept constant within measurements.

### Transmission Electron Microscopy (TEM) and Immuno‐gold EM Analysis

EV Samples were prepared for negative staining as previously described.^[^ ^5^ ^]^ For immuno‐gold EM, 8 µL of isolated EVs were incubated in 2% paraformaldehyde‐0.1 m PBS for 30 min and placed on top of carbon‐coated nickel grids for 15 min. The girds were washed in 0.1 m PBS, blocked in 0.1 m glycine 0.3% BSA for 10 min, and incubated with primary antibodies for 1 and 2 h (Table S3, Supporting Information). Following the primary antibody incubations, grids were blocked for 10 min and incubated with the secondary antibodies with 6 and 15 nm gold particles for 1 h. After washes, a standard negative staining procedure was performed as described above. To observe antibodies binding to the antibody‐binding proteins displayed on EVs, first EVs were incubated with mouse IgG antibodies labeled with gold particles. Afterward, samples were processed as described above. Grids were washed and imaged using an FEI Tecnai G2 Spirit (Thermo Fisher Scientific, Oregon, USA) and a digital camera Morada (Olympus Soft Image Solutions GmbH, Münster, Germany). A standard negative staining procedure using the secondary antibody conjugated to gold nanoparticles was performed to assess unspecific binding.

### Cryo‐Electron Microscopy

Glow discharge technique (15 sec, 7,2 V, using a Bal‐Tec MED 020 Coating System) was applied over Lacey carbon‐coated copper grids. Immediately, 4 µL of the sample was put on the grid and vitrified using an automatic plunge freezer (Leica EM GP, Leica Microsystems Company, Wetzlar, Germany) at 10  °C and 95% humidity. The excess sample was removed by blotting once 2.5 s with filter paper and plunged into liquid ethane at −180 °C. After the vitrification process, the grids were stored immersed in liquid nitrogen until use. The grids were mounted in a Gatan 626 cryo‐holder (Gatan Company, California, USA) and examined using low dose software under a transmission electron microscope FEI Tecnai G2 Spirit BioTwin (ThermoFisher Scientific, Oregon, USA). Pictures were taken using Radius software (version 2.1) with a Xarosa digital camera (EMSIS GmbH, Münster, Germany).

### Single‐Molecule Localization Microscopy (SMLM) Analysis

SMLM analysis was done as described before.^[^ ^7^ ^]^ Briefly, EVs from transfected cells and non‐transfected controls were imaged using a Ti Eclipse inverted microscope (Nikon). EVs were detected as diffraction‐limited objects corresponding to the point spread function of the microscope, confirming the detection of individual vesicles. EV samples from non‐transfected Expi293F cells were used as a negative control for GFP fluorescence background. For the determination of single‐molecule copy number, recombinant GFP (rGFP; SinoBiological) or Alexa Fluor 647‐labeled, or APC‐labeled antibodies (BD) were used as reference. rGFP/Alexa Fluor 647‐Antibody was added to empty wells in 10‐fold dilutions (from 1 nm) and multiple dilutions were imaged using identical settings as above. Identification and quantification of peak intensity by ImageJ from individual fluorophores were achieved using a threshold image (> 2‐fold background intensity). Subsequent time‐resolved imaging of the individual fluorophores was used to confirm the signal originated from single fluorophores, by recording the bleaching traces. Any objects that showed multistep bleaching traces were removed from the analysis. The resulting intensity histogram was used to estimate the average signal of individual fluorophores. Similarly, the intensity histogram of the EVs was used to estimate the average signal, and, by directly dividing it by the fluorophore intensity, the absolute protein copy number could be estimated. Due to the high heterogeneity and lower purity of the EV samples compared to recombinant GFP samples, a more stringent threshold of three GFP molecules was defined as the detection limit of SMLM in our work to provide a more robust analysis.

### Nano‐Flow Cytometry Analysis

Nano‐flow cytometry was performed using the CytoFLEX system (Beckman Coulter, Pasadena, CA) equipped with 3 lasers (405, 488, and 640 nm wavelengths) as previously described.^[^ ^63^ ^]^ The 405 nm violet laser for SSC (V‐SSC) was selected with 1800 manual threshold setting in V‐SSC height channel and 100 of gain of V‐SSC signal in the acquisition setting. Samples were loaded and run at a slow flow rate (10 µl min^−1^) for 1 min until the event/s rate became stable, and then a 20 s acquisition run was saved. Calibrating the Sample Flow Rate was conducted following CytoFLEX Instructions by water weigh difference during 18 min acquisition with slow flow rate. Data were acquired and analyzed using CytExpert 2.0 software (Beckman Coulter) with events/s and events/ml. For events/ml calculation, the background signal of control was subtracted. Percent of the gated region were calculated with the denominator of total events/s. The Gigamix beads are a mixture of an equal volume of fluorescent Megamix‐Plus SSC (BioCytex, France) and Megamix‐Plus FSC beads (BioCytex) which have varied sizes: 100, 160, 200, 240, 300, 500, and 900 nm. For the 100 and 200 nm beads, standard fluorescent polystyrene beads of 100 nm in diameter (NanoSight Ltd., UK) for NTA were used for calibration. To compare the detected particle concentration between NTA and CytoFLEX, standard 100 nm Fluoresbrite YG Microspheres (100 nm Microspheres) were used.

### EV Labeling with HaloTag Ligands, Fluorophore‐Conjugated Antibodies, and Fluorescent Dyes

HaloTag‐containing EVs (10^9^‐10^11^ particles) were conjugated with the following ligands: 1) 5 µM HaloTag TMRDirect Ligand (Promega) for 30 min at 37 °C in the dark; 2) 50 µM Triantennary GalNAc (10 mm Triantennary GalNAc DMSO stock solution was diluted in PBS) for 1 h (for mass‐spectroscopy and GalNAc binding competition assay), for 2 h (for GalNAc‐EVs characterization and binding experiments), 16 h (for mass‐spectroscopy), 24 h (for GalNAc binding competition assay) at RT with very gentle agitation; 3) 50 µM Folate for 1 and 16 h (for mass‐spectroscopy) at RT; 4) Halo protein at 29 µM reacted completely with GE11 peptide (50 µM) within 16 h at RT.

HaloTag‐containing and naïve EVs (10^11^ particles) were labeled with the following antibodies and dye: 1) anti‐Myc Tag Alexa Fluor 488 Antibody (Cell signaling), 2) 6 µM HaloTag Oregon green (Promega), 3) PE anti‐human CD63 Antibody (BioLegend), 4) 250 µm – CellTrace Far Red Cell (Thermo Fisher) for 2 h at RT in the dark. Antibody dilutions are listed in Table S3 (Supporting Information).

To remove the unbound compounds and recover the labeled EVs, samples were added to a size‐exclusion chromatographic resin to separate proteins from small molecules (0.5 mL Zeba Spin Desalting Columns, 40K MWCO (Thermo Scientific)). Excess/free GalNAc was removed using the Zeba Spin Desalting Columns, 40K MWCO (Thermo Scientific), following the manufacturer´s recommendation or density gradient flotation as described in the material and methods, depending on the downstream analysis. The flow‐through sample was collected and analyzed by NTA.

Protein A and unmodified EVs (10^11^‐10^12^ particles) were first incubated with the 100 µL of blocking solution (LI‐COR) for 1 h to avoid unspecific binding of GLP1R antibodies to the EV surface. GLP1R antibodies (Table S3, Supporting Information) were incubated in a blocking solution with EVs for 45 min at RT with shaking. EVs were labeled with 25 µL of 0,2 µg µL^−1^ NHS ester Alexa Fluor 594 dye for 15 min in the solution with antibodies. Subsequently, 1 mL of PBS was added to the samples, and samples were ultra‐centrifugated at 100000 x *g* for 1 h or added to Amicon centrifugal filter units (#UFC503096, Merck) to remove unbound antibodies and dye.

### Cell Binding Assays

To evaluate the binding of EVs to target cells, EVs were engineered to simultaneously display HaloTag at their surface for the clicking of targeting ligands and Nluc protein at their lumen for tracking purposes. Next, 20000 cells well^−1^ were seeded in a white 96 well‐plate 24 h prior to the experiments.

For the cell binding assay in 3D model, pre‐formed primary human hepatocyte spheroids were transferred into Akura 96 Spheroid Microplate (CS‐09‐004‐03, InSphero). For each replicate, an individual spheroid was placed in the Akura plate and incubated with 2,5 × 10^11^ GalNAc or control EVs for 1 h at 4 °C. After incubation, the spheroids were washed with PBS and transferred into a white 96‐well clear bottom plate (Corning) in 0.25% Trypsin‐EDTA solution (Gibco). Before adding Nano‐Glo Assay Reagent, the plate was incubated for 15 min at 37 °C at 700 rpm to dissociate the spheroids for enhanced EV release. The presence of NanoLuc luciferase in cells was measured using the Nano‐Glo Luciferase assay system (N1120, Promega) following the manufacturer´s protocol. Luminescence was measured using the PHERAstar FSX instrument (BMG Labtech). To evaluate binding of anti‐GLP1R antibody‐labeled EVs to HEK293 cells that stably overexpress GLP1R, Protein A‐modified and naïve EVs were labeled with NHS ester Alexa Fluor 594 dye and incubated with antibodies. A concentration of 10^11^–10^12^ particles was added to the cells and incubated at 4 °C for 1 h. Next, unbound EVs were removed, and cells were washed with cold PBS. Alexa Fluor 594 fluorescent signal was measured with the CLARIOstar plate reader (BMG labtech). To evaluate the binding of EVs to unmodified HEK293T cells, Protein A and naive EVs (1011‐1012 particles) were labeled with NHS ester Alexa Fluor 594 dye followed by incubation with the cells and washes as previously described.

### Cell Imaging Analysis

HEK293 cells containing Cre reporter or GAL9 reporter were seeded into 384‐well Cell Carrier Ultra plates (PerkinElmer: 6007558) in complete media a minimum of 16 h before treatment. Hoechst 33 342 was added to the cell culture medium at 0.5 µg mL^−1^ for a minimum of 1 h before imaging experiments to evenly stain nuclei. After this, cell media was removed and replaced directly by media containing control EVs, Cre EVs (both at a concentration of 3.5 × 10^9^ EVs/well), or 0.5 µL Cre Gesicles (Takara) with Chloroquine concentrations 0, 1, 10, 25, 50, 100, or 120 µM. The cell plate was then moved to the microscope and imaged immediately for the time course for the GAL9 assay or 24 h later for the Cre reporter assay. Live‐cell experiments were carried out within a humidified imaging chamber maintained at 5% CO_2_ with a CV7000 (Yokogawa) spinning disk confocal microscope utilizing a 20x objective (NA 0.75). Images were obtained using a 405 nm laser (BP445/45 nm), 488 nm laser (BP522/35), or 561 nm laser (BP600/37) for relevant fluorophores. For microscopy time‐course measurements, the same fields of view were imaged over time that had received the experimental treatment noted in the figure. Images were processed and analyzed for relevant features and parameters indicated in figures utilizing Columbus image‐analysis software (Perkin Elmer, v2.9.1).

### Galectin‐9 (GAL9) Assay

Generation of HEK293‐GAL9 cells and imaging assay was performed as described previously.^[^ ^36^ ^]^ Briefly, HEK293‐GAL9 cells were treated with 3.5 × 10^9^ EVs/well or with 0.5 µL of Cre Gesicles. Spots were identified using maximum intensity projection fluorescence images. Cells were identified using Hoechst 33 342 for nuclei detection and mCherry‐GAL9 for individual cell boundaries. Within individual cell regions of interest, spot populations were quantified using a “Find spots” building block within Columbus software that identifies punctate structures with relative intensities higher than local background cellular intensity, no limits were placed on max puncta size. Data were exported and handled in Spotfire (Tibco, v10.3), in many cases, numerical values obtained were normalized for combining between experimental replicates. Data were exported and plotted with Prism (GraphPad, v9.0.0).

### ELISA Assay on EVs

96‐well plates (Corning) were coated with Poly‐L‐lysine (Sigma) for 4 h at 4 °C. Afterward, plates were washed 3 times with PBS. Vesicles were coated at concentrations of 30, 3, or 0.3 µg per well overnight at 4 °C. After incubation, plates were washed 3 times with PBS and blocked for 1 h in Odyssey TBS Blocking Buffer (LI‐COR Biosciences Inc, Lincoln, NE). Plates were then incubated for 1 h at RT with anti‐Myc IgG1‐HRP antibody (Abcam, 1:50) in a blocking solution. After incubation plates were washed 3 times with PBS. QuantaBlu Fluorogenic Peroxidase Substrate Kit (Thermo Fisher) was used to measure the HRP signal. QuantaBlu solution was incubated for 30 min. The stop solution was kept for 5 min before detecting a signal. A plate reader was used to measure a signal.

### GalNAc‐HaloTag Synthesis

GalNAc Pfp and HaloTag linkers were synthesized according to previously described protocols^[^ ^64^ ^]^ and isolated and stored as the trifluoroacetate (TFA) salt after purification by HPLC (Kromasil column 250 × 50 mm, 10%–70% gradient of acetonitrile over 10 min). It was either used directly or free‐based prior to use with sodium carbonate in methanol.

Triethylamine (0.11 mL, 0.79 mmol) was added to a solution of “GalNAc Pfp” (100 mg, 53 µmol, CAS Registry Number 1684426‐91‐2, see figure for specific structure) and 2‐(2‐((6‐chlorohexyl)oxy)ethoxy)ethan‐1‐amine (58.8 mg, 0.26 mmol, free‐based prior to use as above) in dichloromethane (0.5 mL). The reaction was stirred overnight. The solvent was removed by evaporation and the crude was directly purified by flash column chromatography (Interchim puriflash 4 g column, 3 mL fractions, mobile phase: pure dichloromethane for 5 fractions and then followed by a slow methanol gradient of 1 to 6% in dichloromethane). Product with additional impurities eluted at 2% methanol gradient strength. The relevant fractions were combined, concentrated, and re‐purified under identical conditions. The removal of triethylamine in the first purification caused the product to elute later in the second column, specifically at 10% methanol in dichloromethane (thin layer chromatography mobile phase: 10% methanol in dichloromethane, product Rf = 0.4, visualization with KMnO4). The fractions containing the product – still with some impurities – were combined and the solvent was removed by evaporation. To the resulting material was added a solution of dichloromethane (0.5 mL) and NH3 in methanol (2 M, 1.5 mL). The reaction was stirred at RT overnight (≈16 h). Another portion of NH3 in methanol (4 M, 2 mL) was added and the reaction was allowed to stir at RT for another 16 h. The volatiles were removed by evaporation and the residue dissolved in dimethyl sulfoxide (1 mL) and purified by HPLC (Xbridge column 150 × 19 mm, 5 µm particle size, 30 mL min^−1^ flow, 210 nm detection, 5%–45% gradient of acetonitrile over 16 min, product eluted after 9.5 min). The relevant fractions were combined and freeze‐dried, and the product was obtained as a white solid (35 mg, 42% over two steps).

### Animals

Mouse experiments were approved by the AstraZeneca internal committee for animal studies and the Gothenburg Ethics Committee for Experimental Animals (license number 162–2015+) compliant with EU directives on the protection of animals used for scientific purposes. Male C57BL/6N (Charles River, Sulzfeld, Germany) mice were individually housed in a temperature‐controlled room at 21 °C with a 12:12 h light/dark cycle and controlled humidity (45%–55%). Mice had access to a regular chow diet (R36, Lactamin AB, Stockholm, Sweden) and water ad libitum. Mice were checked daily and weighed weekly.

### In Vivo Toxicity of EVs

A single dose of EVs (1 × 10^11^ particles) or AV5 (1.4 × 10^9^ PFU) was delivered in a 100 µL PBS bolus by tail‐vein injection to 10‐week‐old mice. Control mice received PBS only. Blood was collected from the lateral saphenous vein 6 h after injection into EDTA‐coated tubes (Sarstedt) and kept on ice. Organs were harvested and wet weight was registered. Liver was collected in 4% PFA in 0.1 M phosphate buffer (Histolab) for histological analysis. Liver toxicity was determined by the levels of HAPG, AST, ALT, and ALP in the blood samples (analyzed by Charles River Lab).

### Histology

Slices of the fixed and embedded tissues were stained on Ventana Discovery Ultra system. Slices were incubated with anti F4/80 antibody for macrophage visualization for 1 h. Afterward, a secondary OmniMap anti‐rabbit HRP antibody (Roche) was incubated for 16 min and the signal was detected using a Discovery Teal kit (Roche). Next, an anti‐human CD63 antibody was added for 1 h followed by incubation with OmniMap anti‐rabbit HRP antibody (Roche) for 16 min. Signal was detected with a Discovery purple kit (Roche). Counterstain was done with Hematoxylin II incubated for 4 min.

### Statistical Analysis

All experiments are represented as an average of at least three independent replicates unless indicated in the figure legends. Data was presented as mean ± s.e.m, with an indication of sample size (n) and P value (p<0.05 for significance) for each statistical test described in the figure legends. Statistical analyses were done in R studio software (v 1.4.1106) using paired two‐sided Student's t‐test.

## Conflict of Interest

A.I., L.B., G.O., J.B., E.G., A.G., C.J., M.J.M., S.T., L.V., A.V., J.W., N.D. and E.L.I. current or former employees of AstraZeneca R&D.

## Author Contributions

A.I., J.R., N.D. and E.L.I. conceived and designed the experiments. J.W., N.D. and E.L.I. designed work. A.I., L.B., G.O'D., E.G., A.G., J.B., A.V., M.M., C.S., L.V., C.J., S.T., E.L.I. performed the experiments. A.I. and E.L.I. analyzed the data and prepared the figures. A.I., L.B., J.R., N.D., E.L.I. worked on the manuscript.

## Supporting information

Supporting Information

## Data Availability

The data that support the findings of this study are available in the supplementary material of this article.
