# Supplementary material for: Creating Designer Engineered Extracellular Vesicles for Diverse Ligand Display, Target Recognition, and Controlled Protein Loading and Delivery
Source: Adv Sci (Weinh). 2023 Oct 22;10(34):2304389. doi: 10.1002/advs.202304389 (PMC10700174; doi:10.1002/advs.202304389)
Supplement: Supplementary file 1 — Supporting Information [file ADVS-10-2304389-s001.pdf]

ADVANCED SCIENCE
Open Access

Supporting Information

for *Adv. Sci.*, DOI 10.1002/advs.202304389

Creating Designer Engineered Extracellular Vesicles for Diverse Ligand Display, Target Recognition, and Controlled Protein Loading and Delivery

*Alena Ivanova, Lukas Badertscher, Gwen O'Driscoll, Joakim Bergman, Euan Gordon, Anders Gunnarsson, Camilla Johansson, Michael J. Munson, Cristiana Spinelli, Sara Torstensson, Liisa Vilén, Andrei Voirel, John Wiseman, Janusz Rak, Niek Dekker and Elisa Lázaro-Ibáñez**

Creating Designer Engineered Extracellular Vesicles for Diverse Ligand Display, Target Recognition and Controlled Protein Loading and Delivery

*Alena Ivanova, Lukas Badertscher, Gwen O´Driscoll, Joakim Bergman, Euan Gordon, Anders Gunnarsson, Camilla Johansson, Michael J. Munson, Cristiana Spinelli, Sara Torstensson, Liisa Vilén, Andrei Voirel, John Wiseman, Janusz Rak, Niek Dekker, Elisa Lázaro-Ibáñez**

Supplementary information

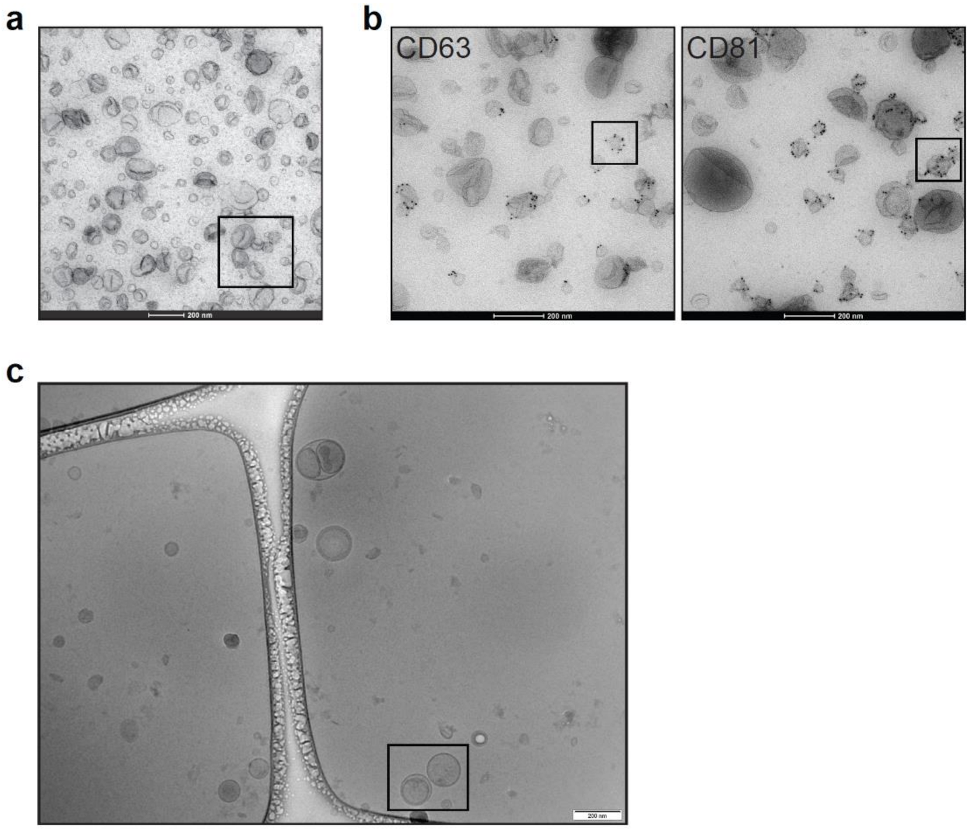

Figure S1. Transmission Electron Microscopy analyses of Expi293F cell-derived EVs.
a) Representative negative staining Transmission Electron Microscopy (TEM) wide-field images of pooled low-density fractions (F1–F3) showing extracellular vesicles (EVs). Scale bar = 200 nm. A zoomed-in section of the image is shown in Fig.1e.
b) Representative immuno-gold negative staining TEM wide-field images of low-density EVs positive for CD63 and CD81 protein markers. Scale bar = 200 nm. Zoomed-in sections for all images are shown in Figure 1e.
c) Cryo-EM image of pooled EV samples (F1–F3) depicting small EV size and morphology. Scale bar = 200 nm. A zoomed-in section of the image is shown in Figure 1f.

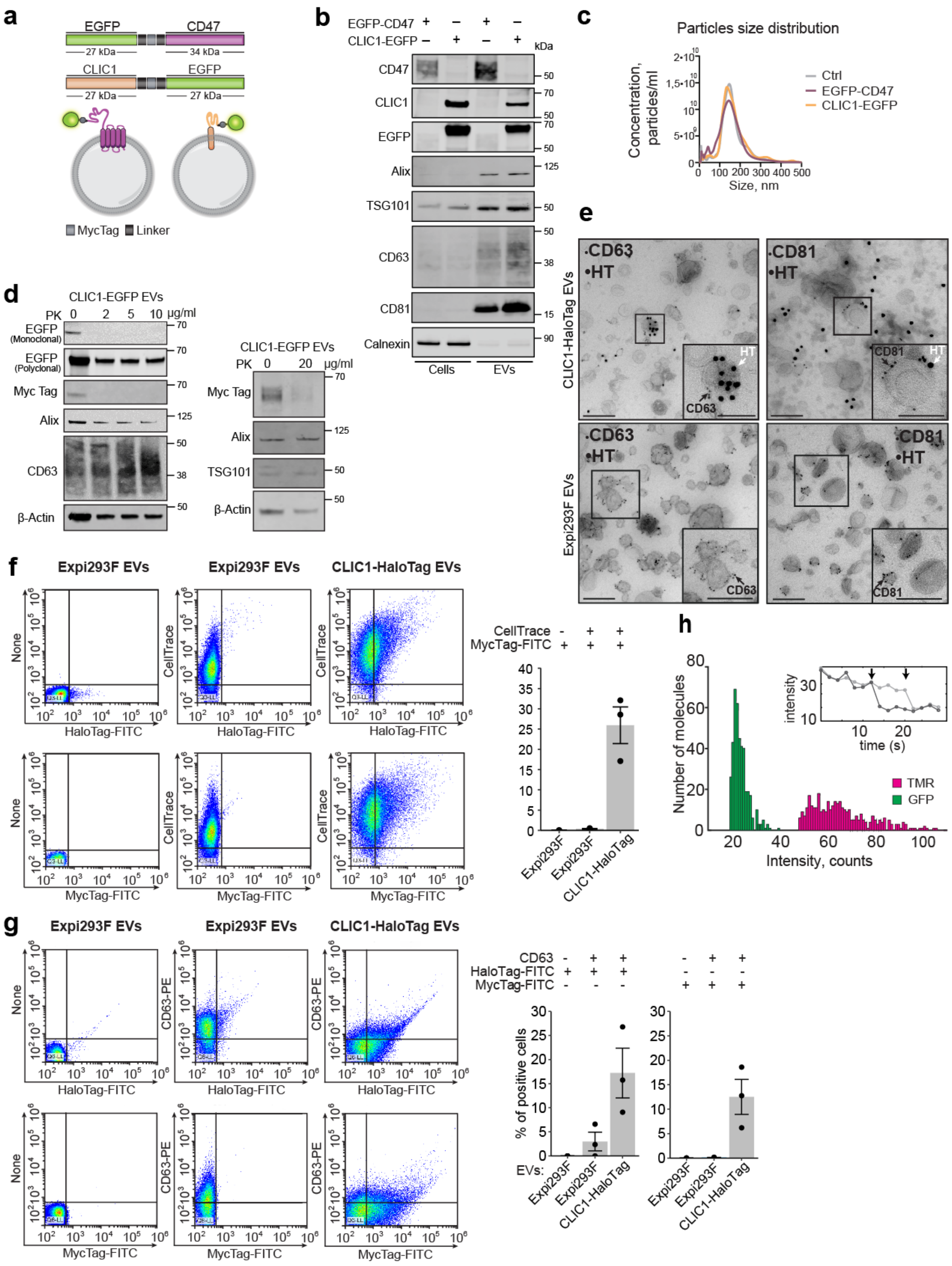

Figure S2. The display of synthetic ligands on the surface of EVs via HaloTag engineering.

a) Schematic representation of the extracellular vesicle (EV) engineering construct design and protein display topology within EVs. Protein names and sizes are denoted.

b) Representative western blot analysis of EVs from EGFP-CD47 and CLIC1-EGFP transfected cells and corresponding cell lysates. Antibodies against protein markers and molecular sizes are shown. The same amount of protein was loaded per lane.
c) Nanoparticle tracking analyses of EVs carrying EGFP-CD47 (purple line), CLIC1-EGFP (orange line) and control naïve EVs (grey line). Representative size distribution and particle concentration graphs are shown.
d) Representative western blot analysis of Expi293F EVs from CLIC1-EGFP transfected cells. Samples were incubated with 2, 5, 10 μg/ml (left blot) or 20 μg/ml (right blot) of Proteinase K (PK) followed by the addition of a protease inhibitor. The same amount of protein was loaded per lane.
e) Immunogold labeling of EVs isolated from Expi293F cells transiently transfected with a plasmid coding for CLIC1-HaloTag and naïve EVs as a negative control. EVs were incubated with primary and secondary antibodies conjugated with 6 nm (for CD63 and CD81) or 15 nm (for HaloTag) gold particles. Representative EV images depict double-positive EVs CD63 (black arrow) and HaloTag (white arrow) or CD81 (black arrow) and HaloTag (white arrow). Scale bars are 200 nm in the wide field and 100 nm in the zoomed-in images.
f) Nano-flow cytometer analysis of Expi293F cell-derived naïve and CLIC1-HaloTag engineered EVs using the CytoFLEX system. HaloTag with Oregon green label and anti-Myc Tag Alexa488 antibody were used for the HaloTag and Myc Tag visualization. The CellTrace Far Red Cell dye was used to label the whole EV fraction. Representative dot plots of CellTrace Far Red Cell versus HaloTag-FITC or Myc Tag-FITC intensity are shown. Quantification of the data is shown in the bottom panels. Results are shown as an average of 3 independent experiments ± s.e.m.
g) Nano-flow cytometer analysis of Expi293F cell-derived naïve and CLIC1-HaloTag engineered EVs. EVs were stained with HaloTag Ligand conjugated with Oregon green dye and anti-Myc Tag Alexa488 antibody. The anti-CD63-PE antibody was used to label CD63 positive EV fraction from the total EV population. Representative dot plots of CD63 versus HaloTag-FITC or Myc Tag-FITC intensity are shown. Results are shown as an average of 3 independent experiments ± s.e.m.
h) Calibration of the single-molecule localization microscopy setup for the quantification of GFP and TMR copy number. Fluorescence intensity distribution for single GFP and TMR molecules. Representative single-step photobleaching profiles (loss of the signal indicated with the arrows) of two GFP molecules are shown in the rectangular.

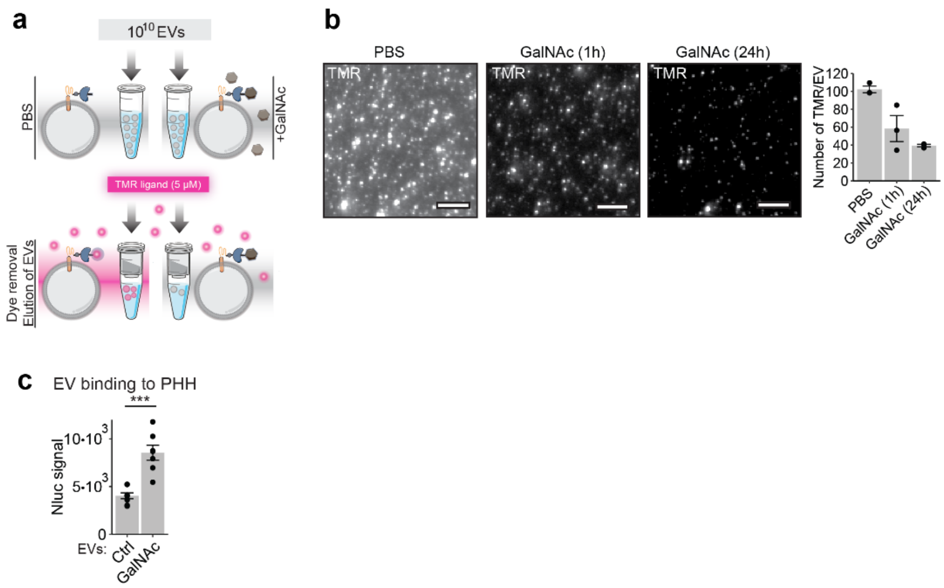

Figure S3. Determination of the number of GalNAc molecules displayed on the surface of EVs.

a) Schematic representation of the ligand competition assay developed to evaluate the number of GalNAc targeting ligands covalently bound to the HaloTag displayed on the EV surface by cell engineering. Synthetic Halo-modified GalNAc ligand (50 μM) or PBS as control (Ctrl) were incubated with HaloTag EVs (10^{10} particles/well) for either 1 hour or 24 hours. Following the initial incubation with GalNAc or PBS, the HaloTag TMR fluorescent ligand (5 μM, shown in pink) was added to the sample and incubated for 30 min at 37°C in the dark. The excess fluorescent ligand was removed by a size-exclusion chromatographic resin and EV samples were analyzed using a multi-well fluorescence microscope to determine the targeting ligand binding efficiency. TMR fluorescent signal indicates the number of HaloTag spots available for binding after incubation with GalNAc targeting ligand.

b) Representative images depicting TMR fluorescent signal in the indicated conditions above. Expi293F cells were transiently transfected with plasmids coding for CLIC1-EGFP-HaloTag. Engineered Expi293F EVs were diluted in PBS and added to a multi-well plate with a glass bottom. EVs were imaged by a total internal reflection fluorescence inverted microscope (TIRF). The plot represents the number of TMR molecules per EV. Incubation with GalNAc for 1 hour or 24 hours reduces the number of TMR molecules bound to HaloTag on EVs. Scale bar = 15 μm.

c) Evaluation of the binding efficiency of engineering EVs displaying GalNAc targeting ligand to primary human hepatocyte (PHH) from the second donor grown in 3D spheroid cultures. A total of 2.5 $x10^{11}$ particles of HaloTag-CLIC1-Nanoluc (Nluc) EVs with and without GalNAc targeting ligand were incubated with primary human hepatocyte spheroids in 96 well plate for 1 hour at 4°C. After incubation, unbound EVs were washed out, and spheroids were lysed to measure the luciferase signal that accounted for EV binding in both targeted (GalNAc) and control (Ctrl) conditions. Eight spheroids per condition were used. Shown is the average of luciferase signals from each spheroid in relative luminescence units ± s.e.m. P-value was calculated using a two-sided Student's T-test. Three asterisks indicate that $p<0.001$.

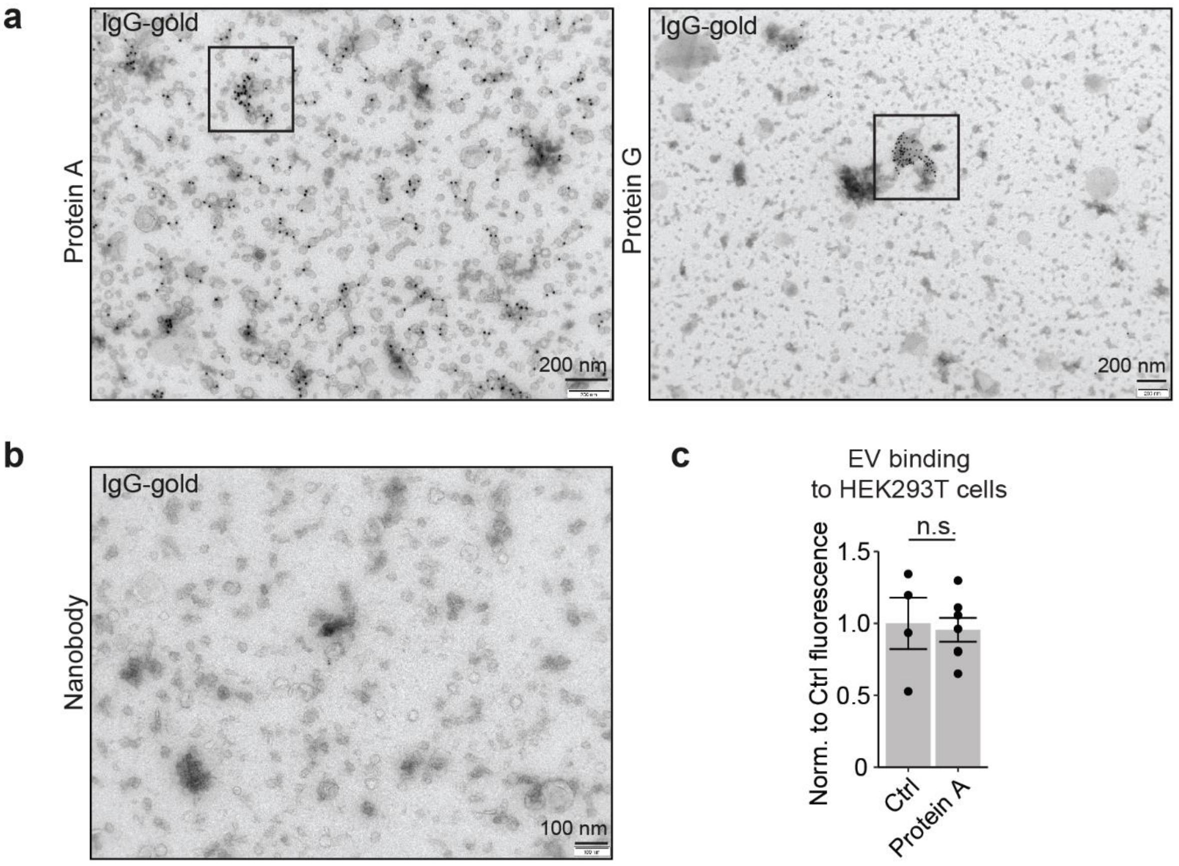

Figure S4. Characterization of EVs carrying antibody-binding proteins by Transmission Electron Microscopy.

a) Representative immuno-gold negative staining transmission electron microscopy (TEM) wide-field images of Protein A-CD81 and Protein G-CD81 EVs incubated with IgG1-gold conjugated antibodies. Scale bar = 200 nm. Zoomed-in sections shown in Figure 3c are denoted with a black box.

b) Representative immuno-gold negative staining TEM wide-field images of Nanobody EVs incubated with IgG1-gold antibodies. Scale bar = 100 nm.

c) Evaluation of the binding efficiency of engineered EVs to HEK293T cells. Protein A EVs or naïve EVs (Ctrl) were labeled with Alexa Fluor 594 NHS Ester dye and incubated with the cells at 4°C. Unbound EVs were washed out, and the normalized fluorescent signal from Ctrl (n=4) and Protein A EVs (n=6) ± s.e.m. is shown. The p-value was calculated using a two-sided Student's T-test. Non-significance (n.s.) indicates that $p>0.05$.

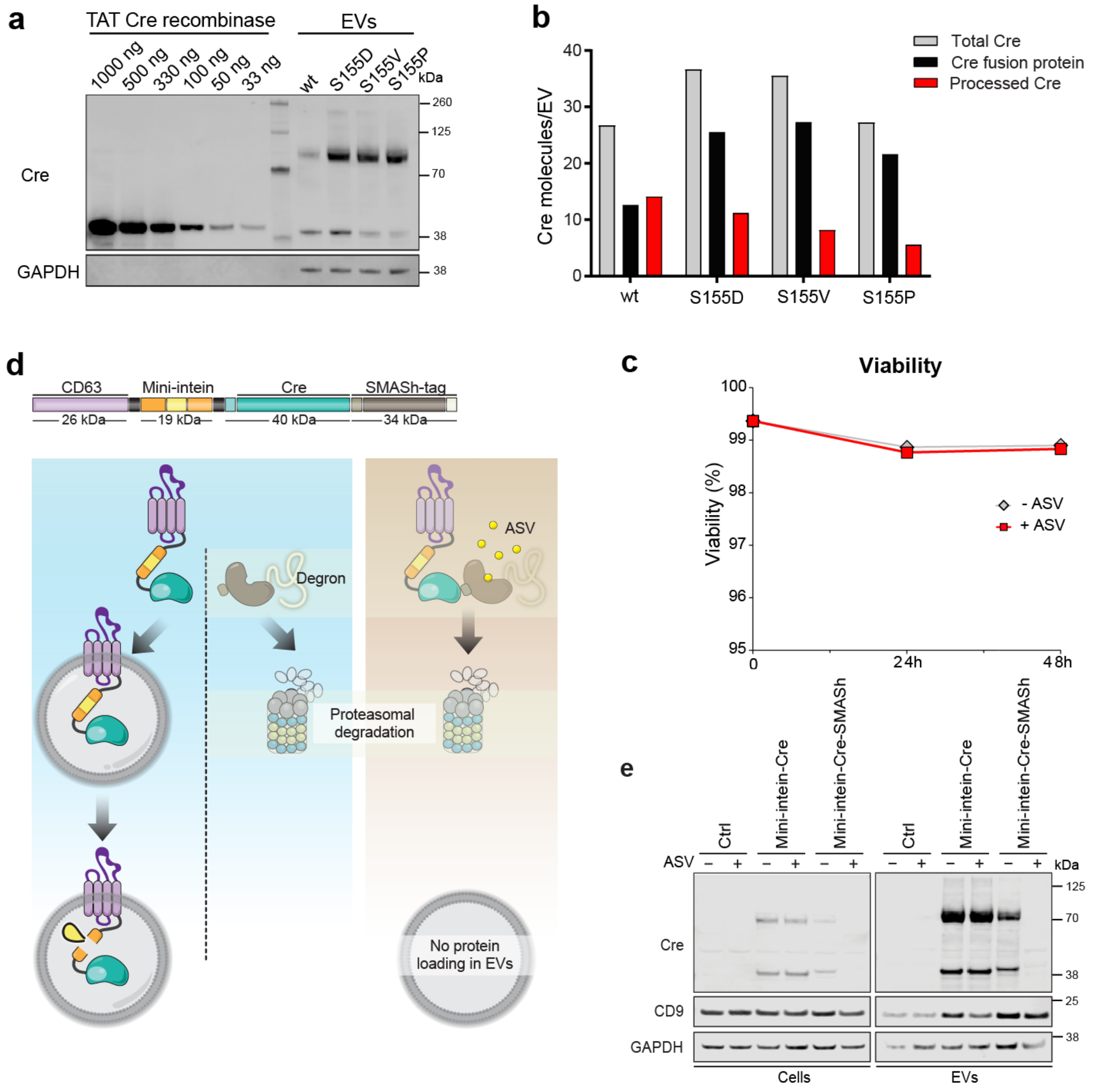

Figure S5. Characterization of the mini-intein and TimeSTAMP EV protein loading systems.

a) Representative western blot analysis of EVs loaded with Cre recombinase (Cre) by the SpDnaB mini intein system. The same number of EV particles were loaded per condition. Cre cargo that is a part of the fusion protein corresponds to a band between 70 and 125 kDa. The cleaved processed Cre was detected around 38 kDa. TAT Cre recombinase was used as a protein standard for quantification purposes. Wild-type (wt., Ser155), Ser substitution for Asp in position 155 (S155D), Ser substitution for Val in position 155 (S155V), and Ser substitution for Pro in position 155 (S155P).

b) Quantification of Cre protein levels loaded in EVs via the SpDnaB mini-intein system. Cre protein levels were evaluated by comparison with a Cre recombinase standard. Cre recombinase fusion protein molecule number was estimated based on the quantification of the western blot band between 70 and 125 kDa intensity. Cleaved Cre recombinase protein molecule number was calculated based on the intensity of the band around 38 kDa. The total Cre recombinase molecule number is a sum of fusion Cre and cleaved Cre protein molecules.

c) Cell viability upon treatment with Asunaprevir (ASV) (3 μM). No significant changes were found between cells treated with ASV (red) and untreated cells (grey). Results are shown as an average of three independent experiments ± s.e.m.

d) Design of a Cre protein loading system with SpDnaB mini-intein domain using the Small Molecule-Assisted Shutoff SMASh-tag, a drug-controllable self-removing degron. (SMASh) tag consists of a degron that removes itself in the absence of the protease inhibitor ASV, leaving untagged protein that is loaded on EVs. In the presence of ASV, the fusion protein keeps the degron tag which leads to its proteasomal degradation.

e) Representative western blot analyses of cells and EVs secreted by Expi293F cells expressing mini-intein or mini-intein-SMASh loading systems with and without ASV.

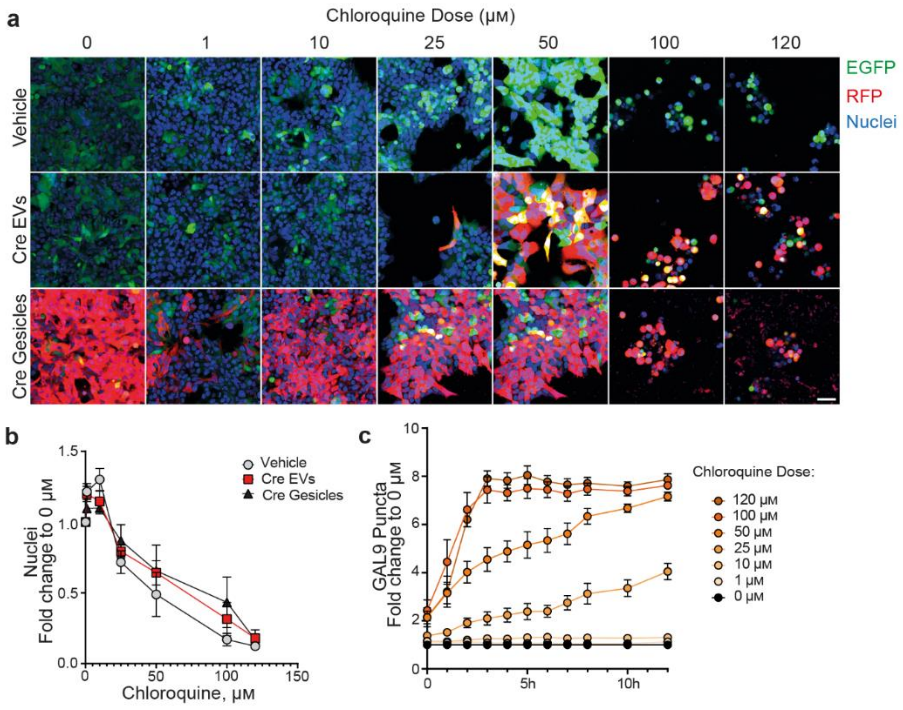

Figure S6. EV cargo delivery is promoted by the endosomal escape enhancer chloroquine.

a) Representative images of the HEK293 Cre-RFP reporter cells upon the treatment with chloroquine and EVs. Cells were treated with indicated series of concentrations of chloroquine together with vehicle, EVs carrying Cre recombinase loaded with mini-intein system (Cre EVs), both at a concentration of 3.5 x 10^9 EVs/well, or 0.5 µl of Cre recombinase Gesicles (Cre Gesicles). Cells were imaged 48 hours after treatment. Scale bar = 50 μm.

b) Quantification of the number of cell nuclei for images acquired in (**a**). Fold-change of nuclei number to 0 μM vehicle control condition is plotted. The average of three independent experiments ± s.e.m. is shown.

c) Quantification of GAL9 puncta in HEK293-GAL9 reporter cells treated with indicated Chloroquine concentrations to identify the optimal concentration for treatment. Cells were imaged at indicated times and quantified for GAL9 puncta. Data represents mean fold-change to the vehicle control from three independent experiments ± s.e.m.

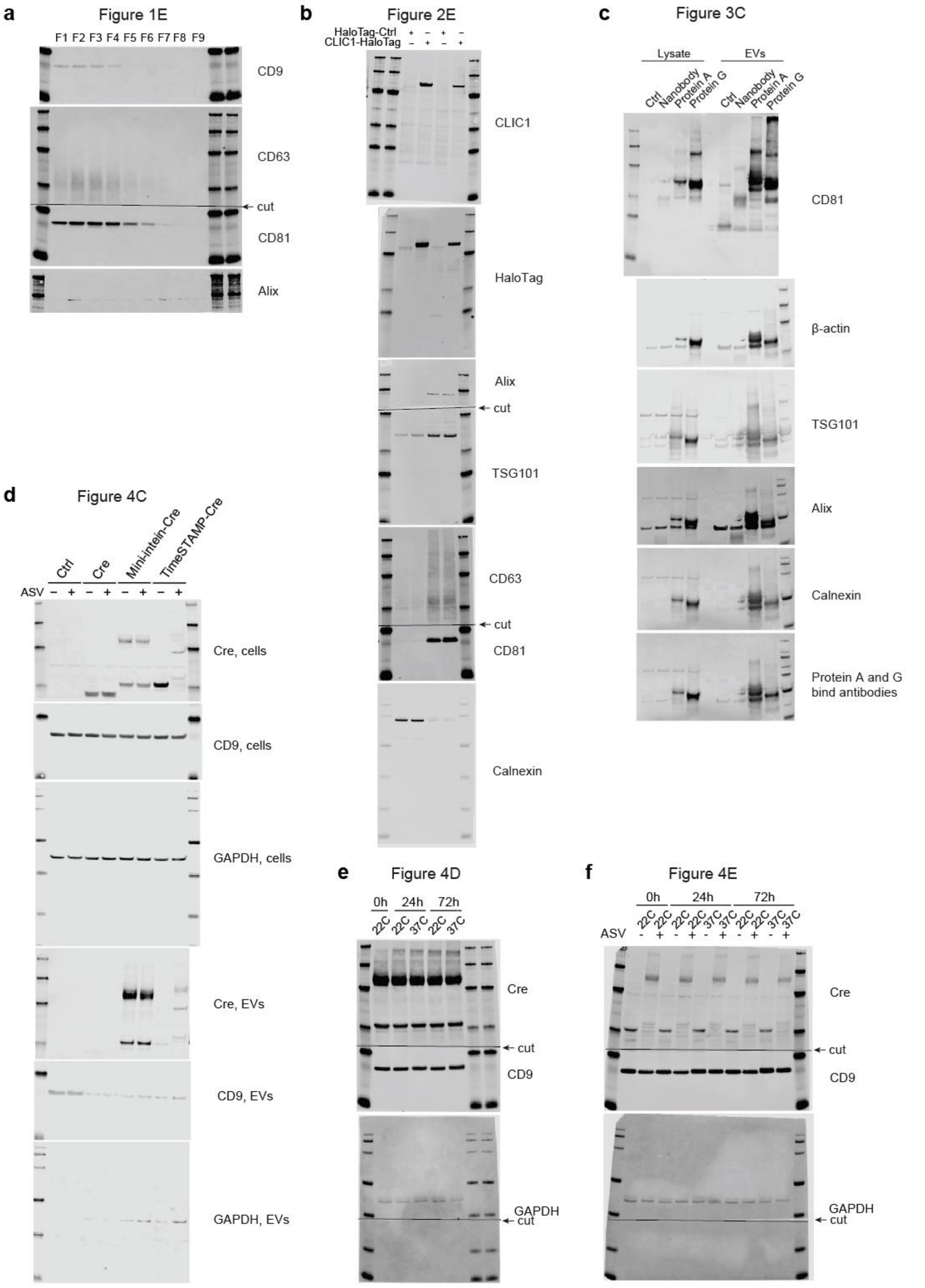

Figure S7. Representative full Western blots.

a-f) Representative full Western blots for indicated Figures.

Supplementary Table 1. Mass spectroscopy.

Sample	Calculated Mass (including N-term Met), MW, Da	Observed Mass, MW, Da	Difference between Calculated and Observed Mass, MW, Da	Comments
HaloTag recombinant protein	34989.02	34990	0.98	Identity confirmed. Clean spectra
Triantennary GalNAc	782.9	1563.8	780.9	Identity confirmed. Clean spectra
Folate	647.6	646.6	-1	Identity confirmed. Clean spectra
HaloTag + GalNAc (1 h incubation)	36552.82	36517.1	-35.72	Identity confirmed. Clean spectra
HaloTag + GalNAc (16 h incubation)	36552.82	36516	-36.82	Identity confirmed. Clean spectra
HaloTag + Folate (1 h incubation)	35635.62	35599.3	-36.32	Identity confirmed. Clean spectra
HaloTag + Folate (16 h incubation)	35635.62	35597.7	-37.92	Identity confirmed. Clean spectra
HaloTag + Alexa Fluor	35988.02	35692.4	-295.62	Identity confirmed.
HaloTag + Oregon Green	35691.02	35612.6	-78.42	Identity confirmed.
HaloTag + GE11 peptide	36946.92	36910.6	-36.32	Identity confirmed. Clean spectra

Supplementary Table 2. Protein sequence of the fusion proteins generated for surface or cargo loading.

N	Protein name	Protein sequence
1	CLIC1-Myc-HaloTag	MAEEQPQVELFVKAGSDGAKIGNCPFSQRLFMVLWLKGVTFNVTTV DTKRRTETVQKLCPGGQLPFLLYGTEVHTDTNKIEEFLEAVLCPPR YPKLAALNPESNTAGLDIFAKFSAYIKNSNPALNDNLEKGLLKALK VLDNYLTSPLPEEVDETSAEDEGVSQRKFLDGNELTLADCNLLPKL HIVQVVCKKYRGFTIPEAFRGVHRYLSNAYAREEFASTCPDDEEIE LAYEQVAKALKGGGGSEQKLISEEDLGGGGSMTPCRALEPTTEDLY FQSDNDGSEIGTGFPFDPHYVEVLGERMHYVDVGPRDGTPVLFLHG NPTSSYVWRNIIPHVAPTHRCIAPDLIGMGKSDKPDLGYFFDDHVR FMDAFIEALGLEEVVLVIHDWGSALGFHWAKRNPERVKGIAFMEFI RPIPTWDEWPEFARETFQAFRTTDVGRKLIIDQNVFIEGTLPMGVV RPLTEVEMDHYREPFLNPVDREPLWRFPNELPIAGEPANIVALVEE YMDWLHQSPVPKLLFWGTPGVLIPPAEAARLAKSLPNCKAVDIGPG LNLLQEDNPDLIGSEIARWLSTLEISG
2	Nluc-CLIC1-Myc-HaloTag	FTLEDFVGDWRQTAGYNLDQVLEQGGVSSLFQNLGVSVTPIQRIVL SGENGLKIDIHVIIPYEGLSGDQMGQIEKIFKVVYPVDDHHFKVIL HYGTLVIDGVTPNMIDYFGRPYEGIAVFDGKKITVTGTLWNGNKII DERLINPDGSLLFRVTINGVTGWRLCERILAGGGGSGGGGSMAEEQ PQVELFVKAGSDGAKIGNCPFSQRLFMVLWLKGVTFNVTTVDTKRR TETVQKLCPGGQLPFLLYGTEVHTDTNKIEEFLEAVLCPPRYPKLA ALNPESNTAGLDIFAKFSAYIKNSNPALNDNLEKGLLKALKVLDNY LTSPLPEEVDETSAEDEGVSQRKFLDGNELTLADCNLLPKLHIVQV VCKKYRGFTIPEAFRGVHRYLSNAYAREEFASTCPDDEEIELAYEQ VAKALKGGGGSEQKLISEEDLGGGGSMTPCRALEPTTEDLYFQSDN DGSEIGTGFPFDPHYVEVLGERMHYVDVGPRDGTPVLFLHGNPTSS

		YVWRNIIPHVAPTHRCIAPDLIGMGKSDKPDLGYFFDDHVRFMDAF<br>IEALGLEEVVLVIHDWGSALGFHWAKRNPERVKGIAFMEFIRPIPT<br>WDEWPEFARETFQAFRTTDVGRKLIIDQNVFIEGTLPMGVVRPLTE<br>VEMDHYREPFLNPVDREPLWRFPNELPIAGEPANIVALVEEYMDWL<br>HQSPVPKLLFWGTPGVLIPPAEAARLAKSLPNCKAVDIGPGLNLLQ<br>EDNPDLIGSEIARWLSTLEISG
3	CLIC1-Myc-EGFP-HaloTag	MAEEQPQVELFVKAGSDGAKIGNCPFSQRLFMVLWLKGVTFNVTTV<br>DTKRRTETVQKLCPGGQLPFLLYGTEVHTDTNKIEEFLEAVLCPPR<br>YPKLAALNPESNTAGLDIFAKFSAYIKNSNPALNDNLEKGLLKALK<br>VLDNYLTSPLPEEVDETSAEDEGVSQRKFLDGNELTLADCNLLPKL<br>HIVQVVCKKYRGFTIPEAFRGVHRYLSNAYAREEFASTCPDDEEIE<br>LAYEQVAKALK<br>GGGGSEQKLISEEDLGGGGSMVSKGEELFTGVVPILVELDGDVNGH<br>KFSVSGEGEGDATYGKLTLKFICTTGKLPVPWPTLVTTLTYGVQCF<br>SRYPDHMKQHDFFKSAMPEGYVQERTIFFKDDGNYKTRAEVKFEGD<br>TLVNRIELKGIDFKEDGNILGHKLEYNYNSHNVYIMADKQKNGIKV<br>NFKIRHNIEDGSVQLADHYQQNTPIGDGPVLLPDNHYLSTQSALSK<br>DPNEKRDHMVLLEFVTAAGITLGMDELYKGGAGGNSRPLEPLELGG<br>GGSMTPCRALEPTTEDLYFQSDNDGSEIGTGFPFDPHYVEVLGERM<br>HYVDVGPRDGTPVLFLHGNPTSSYVWRNIIPHVAPTHRCIAPDLIG<br>MGKSDKPDLGYFFDDHVRFMDAFIEALGLEEVVLVIHDWGSALGFH<br>WAKRNPERVKGIAFMEFIRPIPTWDEWPEFARETFQAFRTTDVGRK<br>LIIDQNVFIEGTLPMGVVRPLTEVEMDHYREPFLNPVDREPLWRFP<br>NELPIAGEPANIVALVEEYMDWLHQSPVPKLLFWGTPGVLIPPAEA<br>ARLAKSLPNCKAVDIGPGLNLLQEDNPDLIGSEIARWLSTLEISG
4	EGFP-Myc-CD47	MPLLLLLPLLWAGALAMVSKGEELFTGVVPILVELDGDVNGHKFSV<br>SGEGEGDATYGKLTLKFICTTGKLPVPWPTLVTTLTYGVQCFSRYP<br>DHMKQHDFFKSAMPEGYVQERTIFFKDDGNYKTRAEVKFEGDTLVN<br>RIELKGIDFKEDGNILGHKLEYNYNSHNVYIMADKQKNGIKVNFKI<br>RHNIEDGSVQLADHYQQNTPIGDGPVLLPDNHYLSTQSALSKDPNE<br>KRDHMVLLEFVTAAGITLGMDELYKGGAGGNSRPLEPLELGGGGSE<br>QKLISEEDLGGGGSMWPLVAALLLGSACCGSAQLLFNKTKSVEFTF<br>CNDTVVIPCFVTNMEAQNTTEVYVKWKFKGRDIYTFDGALNKSTVP<br>TDFSSAKIEVSQLLKGDASLKMDKSDAVSHTGNYTCEVTELTREGE<br>TIIELKYRVVSWFSPNENILIVIFPIFAILFWGQFGIKTLKYRSGG<br>MDEKTIALLVAGLVITVIVIVGAILFVPGEYSLKNATGLGLIVTST<br>GILILLHYYVFSTAIGLTSFVIAILVIQVIAYILAVVGLSLCIAAC<br>IPMHGPLLISGLSILALAQLLGLVYMKFVASNQKTIQPPRKAVEEP<br>LNAFKESKGMMNDE
5	CLIC1-Myc-EGFP	MAEEQPQVELFVKAGSDGAKIGNCPFSQRLFMVLWLKGVTFNVTTV<br>DTKRRTETVQKLCPGGQLPFLLYGTEVHTDTNKIEEFLEAVLCPPR<br>YPKLAALNPESNTAGLDIFAKFSAYIKNSNPALNDNLEKGLLKALK<br>VLDNYLTSPLPEEVDETSAEDEGVSQRKFLDGNELTLADCNLLPKL<br>HIVQVVCKKYRGFTIPEAFRGVHRYLSNAYAREEFASTCPDDEEIE<br>LAYEQVAKALKGGGGSEQKLISEEDLGGGGSMVSKGEELFTGVVPI<br>LVELDGDVNGHKFSVSGEGEGDATYGKLTLKFICTTGKLPVPWPTL<br>VTTLTYGVQCFSRYPDHMKQHDFFKSAMPEGYVQERTIFFKDDGNY<br>KTRAEVKFEGDTLVNRIELKGIDFKEDGNILGHKLEYNYNSHNVYI<br>MADKQKNGIKVNFKIRHNIEDGSVQLADHYQQNTPIGDGPVLLPDN<br>HYLSTQSALSKDPNEKRDHMVLLEFVTAAGITLGMDELYKGGAGGN<br>SRPLEPLEL

6	Nanobody-PGDFR TM-CD81	MPLLLLLPLLWAGALAGGGQVQLVESGGGWVQPGGSLRLS**C**AASGF<br>TFSDTAMMWVRQAPGKGREWVAAIDTGGGYTYYADSVKGRFTISRD<br>NAKNTLYLQMNSLKPEDTARYY**C**AKTYSGNYYSNYTVANYGTTGRG<br>TLVTVSSGGGGSVVISAILALVVLTIISLIILIGGGGSGVEGCTKC<br>IKYLLFVFNFVFWLAGGVILGVALWLRHDPQTTNLLYLELGDKPAP<br>NTFYVGIYILIAVGAVMMFVGFLGCYGAIQESQCLLGTFFTCLVIL<br>FACEVAAGIWGFVNKDQIAKDVKQFYDQALQQAVVDDDANNAKAVV<br>KTFHETLDCCGSSTLTALTTSVLKNNLCPSGSNIISNLFKEDCHQK<br>IDDLFSGKLYLIGIAAIVVAVIMIFEMILSMVLCCGIRNSSVY
7	Protein A-PGDFR TM-CD81	MPLLLLLPLLWAGALAGGGAQHDEAQQNAFYQVLNMPNLNADQRNG<br>FIQSLKDDPSQSANVLGEAQKLNDSQAPKADAQQNKFNKDQQSAFY<br>EILNMPNLNEEQRNGFIQSLKDDPSQSTNVLGEAKKLNESQAPKAD<br>NNFNKEQQNAFYEILNMPNLNEEQRNGFIQSLKDDPSQSANLLAEA<br>KKLNESQAPKADNKFNKEQQNAFYEILHLPNLNEEQRNGFIQSLKD<br>DPSQSANLLAEAKKLNDAQAPKADNKFNKEQQNAFYEILHLPNLTE<br>EQRNGFIQSLKDDPSVSKEILAEAKKLNDAQAPKGGGGSVVISAIL<br>ALVVLTIISLIILIGGGGSGVEGCTKCIKYLLFVFNFVFWLAGGVI<br>LGVALWLRHDPQTTNLLYLELGDKPAPNTFYVGIYILIAVGAVMMF<br>VGFLGCYGAIQESQCLLGTFFTCLVILFACEVAAGIWGFVNKDQIA<br>KDVKQFYDQALQQAVVDDDANNAKAVVKTFHETLDCCGSSTLTALT<br>TSVLKNNLCPSGSNIISNLFKEDCHQKIDDLFSGKLYLIGIAAIVV<br>AVIMIFEMILSMVLCCGIRNSSVY
8	Protein G-PGDFR TM-CD81	MPLLLLLPLLWAGALAGGGLAEAKVLANRELDKYGVSDYYKNLINN<br>AKTVEGVKALIDEILAALPKTDTYKLILNGKTLKGETTTEAVDAAT<br>AEKVFKQYANDNGVDGEWTYDDATKTFTVTEKPEVIDASELTPAVT<br>TYKLVINGKTLKGETTTEAVDAATAEKVFKQYANDNGVDGEWTYDD<br>ATKTFTVTEKPEVIDASELTPAVTTYKLVINGKTLKGETTTKAVDA<br>ETAEKAFKQYANDNGVDGVWTYDDATKTFTVTEGGGGSVVISAILA<br>LVVLTIISLIILIGGGGSGVEGCTKCIKYLLFVFNFVFWLAGGVIL<br>GVALWLRHDPQTTNLLYLELGDKPAPNTFYVGIYILIAVGAVMMFV<br>GFLGCYGAIQESQCLLGTFFTCLVILFACEVAAGIWGFVNKDQIAK<br>DVKQFYDQALQQAVVDDDANNAKAVVKTFHETLDCCGSSTLTALTT<br>SVLKNNLCPSGSNIISNLFKEDCHQKIDDLFSGKLYLIGIAAIVVA<br>VIMIFEMILSMVLCCGIRNSSVY
9	CD63-Mini-intein-NLS-Cre	MAVEGGMKCVKFLLYVLLLAFCACAVGLIAVGVGAQLVLSQTIIQG<br>ATPGSLLPVVIIAVGVFLFLVAFVGCCGACKENYCLMITFAIFLSL<br>IMLVEVAAAIAGYVFRDKVMSEFNNNFRQQMENYPKNNHTASILDR<br>MQADFKCCGAANYTDWEKIPSMSKNRVPDSCCINVTVGCGINFNEK<br>AIHKEGCVEKIGGWLRKNVLVVAAAALGIAFVEVLGIVFACCLVKS<br>IRSGYEVMGGGSGGGSRESGAISGDSLISLASTGKRVSIKDLLDEK<br>DFEIWAINEQTMKLESAKVSRVFCTGKKLVYILKTRLGRTIKATAN<br>HRFLTIDGWKRLDELSLKEHIALPRKLESSSLQLSPEIEKLSQSDI<br>YWDSIVSITETGVEEVFDLTVPGPHNFVANDIIVHN**X**IEQDAGGGS<br>PKKKRKVGGGSMSNLLTVHQNLPALPVDATSDEVRKNLMDMFRDRQ<br>AFSEHTWKMLLSVCRSWAAWCKLNNRKWFPAEPEDVRDYLLYLQAR<br>GLAVKTIQQHLGQLNMLHRRSGLPRPSDSNAVSLVMRRIRKENVDA<br>GERAKQALAFERTDFDQVRSLMENSDRCQDIRNLAFLGIAYNTLLR<br>IAEIARIRVKDISRTDGGRMLIHIGRTKTLVSTAGVEKALSLGVTK<br>LVERWISVSGVADDPNNYLFCRVRKNGVAAPSATSQLSTRALEGIF<br>EATHRLIYGAKDDSGQRYLAWSGHSARVGAARDMARAGVSIPEIMQ<br>AGGWTNVNIVMNYIRNLDSETGAMVRLLEDGD

		**X**= S,D,V or P
10	CD63-TimeSTAMP-NLS-Cre	MAVEGGMKCVKFLLYVLLLAFCACAVGLIAVGVGAQLVLSQTIIQGATPG<br>SLLPVVIIAVGVFLFLVAFVGCCGACKENYCLMITFAIFLSLIMLVEVAA<br>AIAGYVFRDKVMSEFNNNFRQQMENYPKNNHTASILDRMQADFKCCGAAN<br>YTDWEKIPSMSKNRVPDSCCINVTVGCGINFNEKAIHKEGCVEKIGGWLR<br>KNVLVVAAAALGIAFVEVLGIVFACCLVKSIRSGYEVMGGGGSGGGGSED<br>VVCCHSIYGKKKGDIDTYRYIGSSGTGCVVIVGRIVLSGSGTSAPITAYA<br>QQTRGLLGCIITSLTGRDKNQVEGEVQIVSTATQTFLATCINGVCWTVYH<br>GAGTRTIASPKGPVIQMYTNVDQDLVGWPAPQGSRSLTPCTCGSSDLYLV<br>TRHADVIPVRRRGDSRGSLLSPRPISYLKGSSGGPLLCPAGHAVGLFRAA<br>VCTRGVAKAVDFIPVENLETTMRSPVFTDNSSPPAVTLTHPITKIDREVL<br>YQEFDEMEECSQHGGGGSGGGGSPKKKRKVGGGSMSNLLTVHQNLPALPV<br>DATSDEVRKNLMDMFRDRQAFSEHTWKMLLSVCRSWAAWCKLNNRKWFPA<br>EPEDVRDYLLYLQARGLAVKTIQQHLGQLNMLHRRSGLPRPSDSNAVSLV<br>MRRIRKENVDAGERAKQALAFERTDFDQVRSLMENSDRCQDIRNLAFLGI<br>AYNTLLRIAEIARIRVKDISRTDGGRMLIHIGRTKTLVSTAGVEKALSLG<br>VTKLVERWISVSGVADDPNNYLFCRVRKNGVAAPSATSQLSTRALEGIFE<br>ATHRLIYGAKDDSGQRYLAWSGHSARVGAARDMARAGVSIPEIMQAGGWT<br>NVNIVMNYIRNLDSETGAMVRLLEDGD
11	CD63-Mini-intein-NLS-Cre-SMASh tag	MAVEGGMKCVKFLLYVLLLAFCACAVGLIAVGVGAQLVLSQTIIQGATPG<br>SLLPVVIIAVGVFLFLVAFVGCCGACKENYCLMITFAIFLSLIMLVEVAA<br>AIAGYVFRDKVMSEFNNNFRQQMENYPKNNHTASILDRMQADFKCCGAAN<br>YTDWEKIPSMSKNRVPDSCCINVTVGCGINFNEKAIHKEGCVEKIGGWLR<br>KNVLVVAAAALGIAFVEVLGIVFACCLVKSIRSGYEVMGGGSGGGSRESG<br>AISGDSLISLASTGKRVSIKDLLDEKDFEIWAINEQTMKLESAKVSRVFC<br>TGKKLVYILKTRLGRTIKATANHRFLTIDGWKRLDELSLKEHIALPRKLE<br>SSSLQLSPEIEKLSQSDIYWDSIVSITETGVEEVFDLTVPGPHNFVANDI<br>IVHN**D**IEQDAGGGSPKKKRKVGGGSMSNLLTVHQNLPALPVDATSDEVRK<br>NLMDMFRDRQAFSEHTWKMLLSVCRSWAAWCKLNNRKWFPAEPEDVRDYL<br>LYLQARGLAVKTIQQHLGQLNMLHRRSGLPRPSDSNAVSLVMRRIRKENV<br>DAGERAKQALAFERTDFDQVRSLMENSDRCQDIRNLAFLGIAYNTLLRIA<br>EIARIRVKDISRTDGGRMLIHIGRTKTLVSTAGVEKALSLGVTKLVERWI<br>SVSGVADDPNNYLFCRVRKNGVAAPSATSQLSTRALEGIFEATHRLIYGA<br>KDDSGQRYLAWSGHSARVGAARDMARAGVSIPEIMQAGGWTNVNIVMNYI<br>RNLDSETGAMVRLLEDGDGGGGSDEMEECSQHLPGAGSSGDIMDYKDDDD<br>KGSSGTGSGSGTSAPITAYAQQTRGLLGCIITSLTGRDKNQVEGEVQIVS<br>TATQTFLATCINGVCWAVYHGAGTRTIASPKGPVIQMYTNVDQDLVGWPA<br>PQGSRSLTPCTCGSSDLYLVTRHADVIPVRRRGDSRGSLLSPRPISYLKG<br>SSGGPLLCPAGHAVGLFRAAVCTRGVAKAVDFIPVENLETTMRSPVFTDN<br>SSPPAVTLTHPITKIDTKYIMTCMSADLEVVTSTWVLVGGVLAALAAYCL<br>STGCVVIVGRIVLSGKPAIIPDREVLY

Supplementary Table 3. Antibody list.

N	Reagent	Source	Identifier	Comments
1	CLIC1	Abcam	ab76592	Application WB, dilution 1:1000
2	HaloTag	Promega	G9281	Application WB, dilution 1:1000; Immuno EM, dilution 1:50
3	Alix	Abcam	ab117600	Application WB, dilution 1:1000

4	TSG101	Abcam	ab30871	Application WB, dilution 1:1000
5	CD81	Abcam	ab79559	Application WB, dilution 1:1000; Immuno EM, dilution 1:50
6	CD63	Abcam	ab59479	Application WB, dilution 1:1000; Immuno EM, dilution 1:50
7	Calnexin	Abcam	ab22595	Application WB, dilution 1:1000
8	EGFP	Abcam	ab13970	Application WB, dilution 1:1000
9	EGFP	Sigma	GSN149	Application WB, dilution 1:1000
10	Myc	Abcam	ab32	Application WB, dilution 1:1000
11	b-Actin	Sigma-Aldrich	A1978	Application WB, dilution 1:2000
12	CD47	Biorbyt	orb39828	Application WB, dilution 1:1000
13	Cre	Millipore	MAB3120	Application WB, dilution 1:1000
14	GAPDH	Cell signaling	2118	Application WB, dilution 1:1000
15	CD9	Abcam	ab97999	Application WB, dilution 1:1000
16	Goat anti Mouse 680	Licor	925-68070	Application WB, dilution 1:20000
17	Goat anti Mouse 800	Licor	926-32210	Application WB, dilution 1:20000
18	Goat anti Rabbit 680	Licor	926-68071	Application WB, dilution 1:20000
19	Goat anti Rabbit 800	Licor	926-32211	Application WB, dilution 1:20000
20	Donkey anti chicken 800	Licor	925-32218	Application WB, dilution 1:20000
21	Alexa Fluor 647 Mouse IgG2b	BD	558713	Application IF, dilution 1:50
22	Alexa Fluor 647 Mouse IgG2a	BD	558053	Application IF, dilution 1:50
23	APC Mouse IgG1	BD	550854	Application IF, dilution 1:50
24	APC Mouse IgG3	BD	IC007A	Application IF, dilution 1:50
25	Myc-HRP	Abcam	ab62928	Application ELISA, dilution 1:50
26	GLP1R	Developmental Studies Hybridoma Bank	Mab7F38	Application binding assay, dilution 1:50
27	F4/80	Cell signaling	D2S9R	Application binding assay, dilution 1:500
28	CD63	US Biological	033528	Application binding assay, dilution 1:300
29	Myc Tag Alexa Fluor 488	Cell signaling	2279	Application Nano Flow
30	PE anti-human CD63	BioLegend	353004	Application Nano Flow
31	F4/80	Cell Signaling	70076	Application IHC
32	OmniMap anti-rabbit HRP	Roche	760-311	Application IHC

WB: western blot; EM: electron microscopy; IF: immunofluorescence; HRP: Horseradish peroxidase

Supplementary Methods

Halo protein expression and purification

The coding sequence for HaloTag (from Promega vector pH6HTN His6HaloTag T7 Vector) was cloned behind an N-terminal hexahistidine tag into pET24 for expression in E. coli BL21-Star (DE3) (Invitrogen #44-0049). A single colony from Lb/Kan plate was grown at a 1 L scale in Tb/auto-induction medium[1] supplemented with kanamycin for 40 hours at 20ºC. Cells were collected by centrifugation (35 g wet weight) and resuspended using a thorax mixer in 200 ml ice-cold lysis buffer (50 mM Hepes pH 7.5, 150 mM NaCl, 1 mM TCEP and complete protease inhibitors (Roche 11836153001, Sigma Aldrich). Cells were lysed by a single pass on a Constant System at 20 kpsi. The lysate was centrifuged for 15 min at 8000 rpm in a JLA10.500. Imidazole was added to the supernatant to a final concentration of 20 mM to reduce non-specific background binding and 10 ml of a 50% slurry of NTA super flow beads was added. Binding was allowed for 2 hours on a rotating platform at 4ºC. The resin was collected and poured into a 25 ml column and washed using 50 ml lysis buffer. HaloTag protein was eluted using 25 ml of 200 ml imidazole in lysis buffer. Purified protein was dialyzed against 50 mM Hepes pH 7.5/150 mM NaCl, 0.1 mM TCEP at 4ºC. The final sample volume was 23 ml. Absorbance was measured at 280 nm and protein concentration was calculated using Abs (0.0.1 %) of 1.67 providing a value of 11 mg/ml, corresponding to a total of 255 mg of purified Halo protein from 1 L of culture. Protein was aliquoted and flash frozen for storage at - 80ºC. SDS-PAGE analysis showed a single band at the expected position with purity greater than 95 %. LCMS analysis gave a measured mass of 34990 Da, which was within 1 Da of the calculated mass of 34989 Da.

Folate-HaloTag and GE11 peptide-HaloTag synthesis

To a solution of folic acid (225 mg, 0.51 mmol) (commercially available from Enamine) in dry DMSO (5 ml, Sigma Aldrich Sure-seal) in an oven-dried 25 ml 2-neck flask was added diisopropylcarbodiimide (DIC, 80 μl, 0.51 mmol). The mixture was stirred vigorously in darkness, making sure that everything had dissolved. Slight heating was applied with a heat gun if necessary to dissolve all components. After 1.5 hours, a solution of the trifluoroacetate (TFA) salt of “HaloTag Linker” 2-(2-((6-chlorohexyl)oxy)ethoxy)ethan-1-amine (76 mg, 0.34 mmol) in 1 ml DMSO was added followed by triethylamine (142 μl, 1.02 mmol) and the reaction was monitored by LCMS. Reaction was purified directly after 40 hours. The reaction was set-up in duplicate and purified 2 ml at once by HPLC (Kromasil column 250 x 50 mm, 30 mL/min flow, 15-65 % gradient of acetonitrile over 40 min, TFAH buffer). Both the α- and γ- isomers are observed and their separation was difficult to achieve; however practical quantities of desired α-isomer product could be obtained resulting in 18.7 mg of Folate-HaloTag as an orange solid after freeze drying of the fractions containing only desired regioisomer.

GE11 peptides (GenScript) were synthesized with an amidation as a C-Terminal modification. To prepare peptide-HaloTag conjugates, each peptide was incubated with EVs-containing HaloTag or purified HaloTag protein overnight at room temperature in PBS.

References

[1] F. W. Studier, *Methods Mol Biol*, 2014, **1091**, 17-32.
